# Home Life: Factors Structuring the Bacterial Diversity Found within and between Homes

**DOI:** 10.1371/journal.pone.0064133

**Published:** 2013-05-22

**Authors:** Robert R. Dunn, Noah Fierer, Jessica B. Henley, Jonathan W. Leff, Holly L. Menninger

**Affiliations:** 1 Department of Biology and Keck Center for Behavioral Biology, North Carolina State University, Raleigh, North Carolina, United States of America; 2 Cooperative Institute for Research in Environmental Sciences, University of Colorado, Boulder, Colorado, United States of America; 3 Department of Ecology and Evolutionary Biology, University of Colorado, Boulder, Colorado, United States of America; Uppsala University, Sweden

## Abstract

Most of our time is spent indoors where we are exposed to a wide array of different microorganisms living on surfaces and in the air of our homes. Despite their ubiquity and abundance, we have a limited understanding of the microbial diversity found within homes and how the composition and diversity of microbial communities change across different locations within the home. Here we examined the diversity of bacterial communities found in nine distinct locations within each of forty homes in the Raleigh-Durham area of North Carolina, USA, using high-throughput sequencing of the bacterial 16S rRNA gene. We found that each of the sampled locations harbored bacterial communities that were distinct from one another with surfaces that are regularly cleaned typically harboring lower levels of diversity than surfaces that are cleaned infrequently. These location-specific differences in bacterial communities could be directly related to usage patterns and differences in the likely sources of bacteria dispersed onto these locations. Finally, we examined whether the variability across homes in bacterial diversity could be attributed to outdoor environmental factors, indoor habitat structure, or the occupants of the home. We found that the presence of dogs had a significant effect on bacterial community composition in multiple locations within homes as the homes occupied by dogs harbored more diverse communities and higher relative abundances of dog-associated bacterial taxa. Furthermore, we found a significant correlation between the types of bacteria deposited on surfaces outside the home and those found inside the home, highlighting that microbes from outside the home can have a direct effect on the microbial communities living on surfaces within our homes. Together this work provides the first comprehensive analysis of the microbial communities found in the home and the factors that shape the structure of these communities both within and between homes.

*“Man is an animal suspended in webs of significance he himself has spun.” ∼ Clifford Geertz*

## Introduction

Most primates modify the environment in which they live, but humans are unique in producing elaborate homes, structures that through time have become more discrete and isolated from the outdoor environment [1]. In these modern indoor environments we have become isolated from conspicuous life and yet remain surrounded by other species including microbes, fungi, arthropods, and plants. These are now the species with which we interact most frequently and those that are most likely to influence our health and well-being. This is particularly true for bacteria and fungi that live in our homes. These taxa can have both positive and negative effects on human health [2]–[5]. They also produce most household odors [6], [7], are capable of degrading building materials [8], [9], and can contaminate heating/cooling systems within homes [10]. Despite their potential importance, we know surprisingly little about the composition of the microbial communities living in our homes and how the structure of house-associated microbial communities changes across specific locations (habitats) within homes or within the same location across homes [11].

While culture-based studies of microbes in homes tend to focus on selected taxa, including those pathogenic bacteria found in kitchens or bathrooms [12]–[15], house-associated microbial communities are ubiquitous and diverse. This diversity includes some potential pathogens but also many hundreds or even thousands of other taxa that can be identified within homes. The question is no longer whether a given household habitat plays host to bacteria but rather how many and what kind. Diverse microbial communities have been found in bathrooms [16] (including showers [17], [18]), kitchens [19] and indoor air [20]. We know far less about how microbial communities vary across the wide range of surfaces found within homes, surfaces that can represent a broad range of environmental conditions. Cleaning frequencies, surface materials, ventilation rates, the origin of the microbial inocula, and chemical exposure all vary among habitats within houses. Given this great variation within the home, one might expect the microbial taxa found in different habitats to be very different.

Studies of individual bacterial taxa, such as species of the genus *Pseudomonas*, have identified key differences between the strains present in kitchen sinks and bathroom sinks [21]. With the availability of culture-independent molecular approaches for surveying microbial diversity, we are now able to explore how the overall structure of bacterial communities varies across surfaces within homes. For example, recent work by Flores et al. [19] has demonstrated that bacterial communities found on different kitchen surfaces are distinct and that many of these differences are driven by usage patterns and differences in the available sources of microbes among habitats. Similarly, Kelley et al. [18] studied shower curtains and found microbial taxa that seem to be rare elsewhere in homes. To our knowledge, direct and comprehensive comparisons of the microbial composition in other household environments with which we come into contact on a regular basis have yet to be made.

In addition to variation among habitats within houses, we also expect bacterial communities to vary in composition between homes. Globally, homes vary from simple stick structures similar in their climate to the outdoor environment to climate-invariant mansions. But even within regions, house conditions can vary in subtler but important ways, and it is likely that microbial communities also vary among such houses. Such variation could be stochastic–a function of the complex dynamics influencing the fate of the many species and their populations among which we live. Alternately, composition might be influenced deterministically by differences between homes in outdoor or indoor environmental conditions, surface materials, and the inhabitants (both human and otherwise) of the homes. Both indoor humidity and temperature appear to influence the diversity and composition of building-associated bacterial communities [22], [23]. Likewise, the size and cleaning frequency of homes might be expected to influence microbial composition [14] as could the ventilation rate [24], [25], the number of occupants in the home [26], or the specific materials used on a given building surface [27]. It is also possible that the presence of dogs, cats and other domestic animals could influence house-associated microbial communities, as has been suggested previously [28]. There are clearly a multitude of factors that could influence microbial diversity within homes, but the relative importance of these various factors has yet to be adequately determined.

To understand the differences in bacterial communities found across specific house locations (habitats) within homes and the variation that exists within habitats between homes, we sampled bacteria in nine standardized locations within each of forty homes in the Raleigh-Durham area of North Carolina. The sampled homes spanned a range of designs, sizes, numbers and types of occupants (both human and animal occupants), degrees of urbanization, and socioeconomic status. They were identified as part of a volunteer-based citizen science project in which participants sampled their own homes. We compared the diversity and composition of the bacterial communities in the 360 collected samples using high-throughput sequencing of the bacterial 16S rRNA gene.

## Methods

Historically, most efforts to engage the public in scientific research have rallied participation around charismatic organisms or easily observable environmental phenomena [29]. With rapid advances in sequencing technology, associated declines in sequencing costs, and increased public interest in microbiomes, we are now able to engage citizens directly in the study of the “invisible” species with whom they share their daily lives. Building on earlier successes in citizen microbiology (see Belly Button Biodiversity, [30]), we launched the Wild Life of Our Homes (WLOH) project: (http://www.yourwildlife.org/projects/wild-life-of-our-homes/).

Here we present the results from the first forty households we recruited to participate in our study in North Carolina in autumn 2011. Each participant was provided with a written Informed Consent form approved by the North Carolina State University’s Human Research Committee (Approval No. 2177) as well as instructions for sampling their home and a home microbe sampling kit.

Each home sampling kit contained nine dual-tipped sterile BBL™ CultureSwabs™. Participants were instructed to sample nine standardized locations within their home: kitchen cutting board, kitchen counter, a shelf inside a refrigerator, toilet seat, pillowcase, exterior handle of the main door into the house, television screen, the upper door trim on the outside surface of an exterior door, and the upper door trim on an interior door (Figure S1). These locations were selected because they are readily identifiable, they exist in nearly all homes, and we expected them to harbor distinct communities due to differences in usage patterns and/or the likely inputs of bacteria to that surface. The door trims were selected as sampling locations because they are unlikely to be cleaned frequently and they should serve as passive collectors of outdoor or indoor aerosols with little to no direct contact from the home occupants.

## Molecular Analyses

We used the direct PCR approach described in Flores et al. [31] to amplify bacterial DNA from each of the 360 swabs (plus appropriate negative controls). This approach allows the diversity and composition of Operational Taxonomic Units (OTUs) in each sample site to be estimated though it does not distinguish between living and recently dead microbes. Briefly, swab tips were cut into wells in 2 mL 96-well deep well plates and processed using the Extract-N-Amp Plant PCR kit (Sigma-Aldrich, Inc) with modified reagent volumes. The V4/V5 region of the 16S rRNA gene was amplified from each DNA sample in triplicate using reaction conditions and the 515f/806r primer pair described previously [31]. Amplicons from the triplicate reactions were pooled together, quantified using a PicoGreen dsDNA assay, and the amplicons from the 360 samples were pooled together in equimolar concentrations for sequencing on either an Illumina HiSeq or MiSeq instrument. Details on the sequencing approach are available in Caporaso et al. [32]. We obtained a total of 65 million reads that were trimmed to 89 bp in length; only the forward reads were used for downstream analyses as per Caporaso et al. [32]. While there are questions for which 89 bp is insufficient, it has been repeatedly shown that short amplicon reads suffice for accurate taxonomic/phylogenetic placement of sequences [33]. Of the 360 samples, 38 samples did not yield a sufficient number of reads for downstream analysis. Sequence data were processed using QIIME [34] with phylotypes picked at the ≥97% similarity level using the closed reference-based protocol with UCLUST and the Greengenes reference database [35]. The selection of ≥97% similarity level is standard bioinformatics practice for studies using high-throughput sequence data to describe microbial communities [32], [36], [37]; this similarity level is chosen to group sequences together that represent closely-related lineages, not to describe 'species-level' variability, and thus reduces the complexity of the dataset and allows for downstream analyses.

Singletons and phylotypes classified as chloroplasts were removed from further analyses. Phylotypes observed in extraction controls were removed as per Flores et al. [31]. OTUs were considered to be contaminants if they were present in at least 25% of control samples from either sequencing run (Table S1). After removing contaminant sequences and chloroplasts, the number of quality-filtered reads per sample was between 7 and 24,783 (median = 1619.5).

Data have now been deposited online at figshare (http://dx.doi.org/10.6084/m9.figshare.674588).

## Data Analyses

To compare all samples at an equivalent sequencing depth, we randomly selected 1000 reads per sample, and this rarefied dataset was used for all downstream analyses. A number of studies show that alpha and beta diversity patterns are robust to sequencing depth and that 1000 sequences per sample should be more than sufficient to describe overall patterns in alpha diversity and describe changes in community composition across samples, beta diversity (see [38]). Our overall objective was not to determine the full-extent of microbial diversity in a given sample or home (which has, to our knowledge, yet to be accomplished for any sample in any home, much less an entire home), but to compare differences in relative diversity levels and community composition across all of the samples collected for this study.

To determine if alpha diversity, the number of bacterial phylotypes identified out of the 1000 reads per sample, was significantly different among homes or locations within homes, we used a Kruskal-Wallis test. In order to assess differences in the composition of the bacterial communities among homes and locations within homes, we used a phylogenetic metric of community similarity, unweighted UniFrac distance [39], which provided qualitatively similar results to those obtained using the weighted UniFrac distances. The resulting distance matrices were visualized using principal coordinates analyses. In considering differences among habitats within homes, our goal was to assess whether such differences exist and whether they are predictable. The statistical significance of differences in community composition among sampling locations and homes was determined using PERMANOVA with sampling location as a fixed effect and home as a random effect. PERMANOVA was also used to assess the potential influence of other factors in driving differences in community composition among houses by including each key factor as a fixed effect along with sampling location and home (as above) in separate tests. The factors considered included seven self-reported measures of the domestic biome: number of human occupants, presence of children, presence of dogs, presence of cats, recent use of pesticides, presence of carpet, and the presence of and types of allergies. All factors were treated as categorical variables. In considering the influence of pets, we focused on the presence of dogs. Thirteen of the houses we sampled had only dogs as pets and the influence of dogs is likely to be greater than that of other indoor domestic animals because of their large size and need to go outdoors. It would be interesting to consider other domestic animals as well, in particular cats. However, only three houses among those we studied had cats but not dogs (three houses had both). We assessed the significance of the relationship of interior and exterior door trim microbial community composition using a Mantel test. Principal coordinates analysis, PERMANOVA tests, cluster analysis, and Mantel tests were performed using PRIMER 6 [40].

In order to identify the relative importance of different potential source environments to the bacterial communities found on the sampled surfaces we used a source-tracking approach similar to that described previously [19], in which samples from various potential sources are formally compared to those in different study habitats. Briefly, lists of human-associated source taxa were derived from skin, oral cavity, and stool samples, respectively [41]. Leaf taxa were derived from several tree species, lettuce, and spinach [19], [42], and soil source taxa were derived from a diverse array of 88 different soils [43]. We recognize that there may be other potential sources of bacteria in the home environment that we did not consider, but we chose these sources as they are likely to be the most important sources of bacteria found inside or outside homes [16], [19], [25]. Source 16S rRNA gene sequence datasets were processed in QIIME [34] using open reference-based OTU picking against the Greengenes reference database [35]. Source samples were rarefied at 250 sequences per sample prior to further analysis. Indicator taxa were determined using indicator species analysis [44] as implemented by the function, indval (http://CRAN.R-project.org/package=labdsv) in R [45]. Only taxa with an indicator value >0.6 (which is to say, taxa that were highly associated with a particular environment) were used as indicator taxa (Table S2). With one exception (the phylum Acidobacteria), indicator taxa analyses were conducted with taxa grouped at the family level of taxonomic resolution.

## Results

### Variation in Community Structure across Locations within Homes

**Figure 1:**
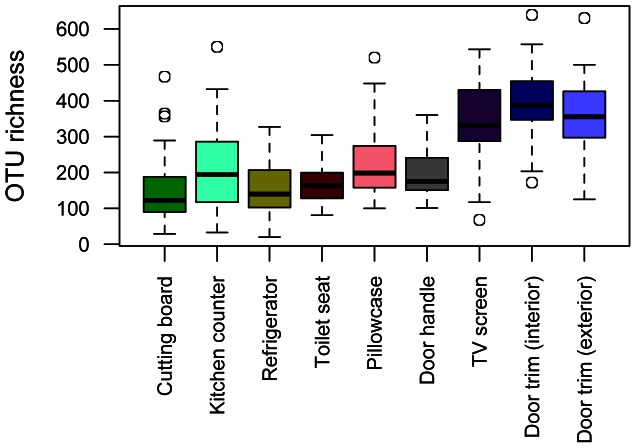
Differences in mean OTU richness across the locations sampled in each of the 40 homes. OTU richness was calculated as the number of bacterial phylotypes identified out of 1000 reads per sample. Boxes represent 95% confidence intervals. Bars denote minimum and maximum values excluding outliers.

Across all samples, a total of 7726 unique phylotypes were identified with nearly all individual samples having at least 100 unique OTUs (out of 1000 reads per sample) (Figure 1). We observed an average of 2253 unique OTUs per home (range 1547 to 3360) when combining all nine samples from the within-home sites. We compared diversity levels across the nine sampling locations and found that, alpha diversity, the number of phylotypes per sample, was consistently highest on those sampling locations where aerosols and airborne particulates seem more likely to collect whether inside (TV screen, interior door trim) or outside the home (exterior door trim) (Figure 1).

**Figure 2:**
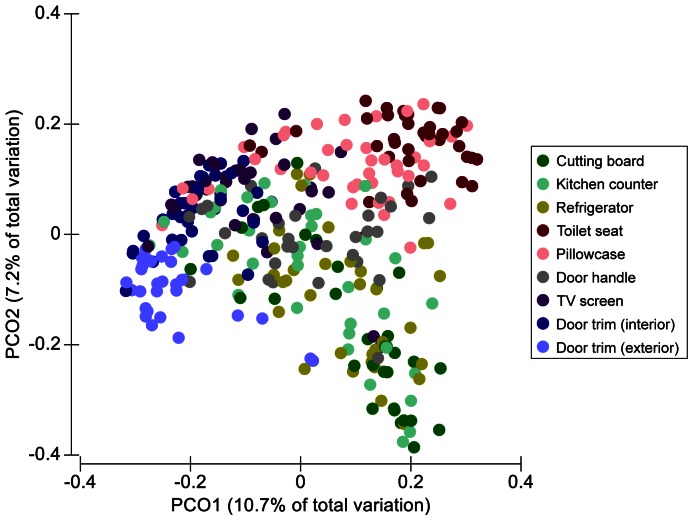
Principal coordinates plot showing overall variation in bacterial community composition among habitats and homes. Differences in the composition of the bacterial communities were quantified using the unweighted UniFrac distance metric. Symbols are colored by habitat (sample location) with symbols closer together representing samples with more similar bacterial communities.

**Figure 3:**
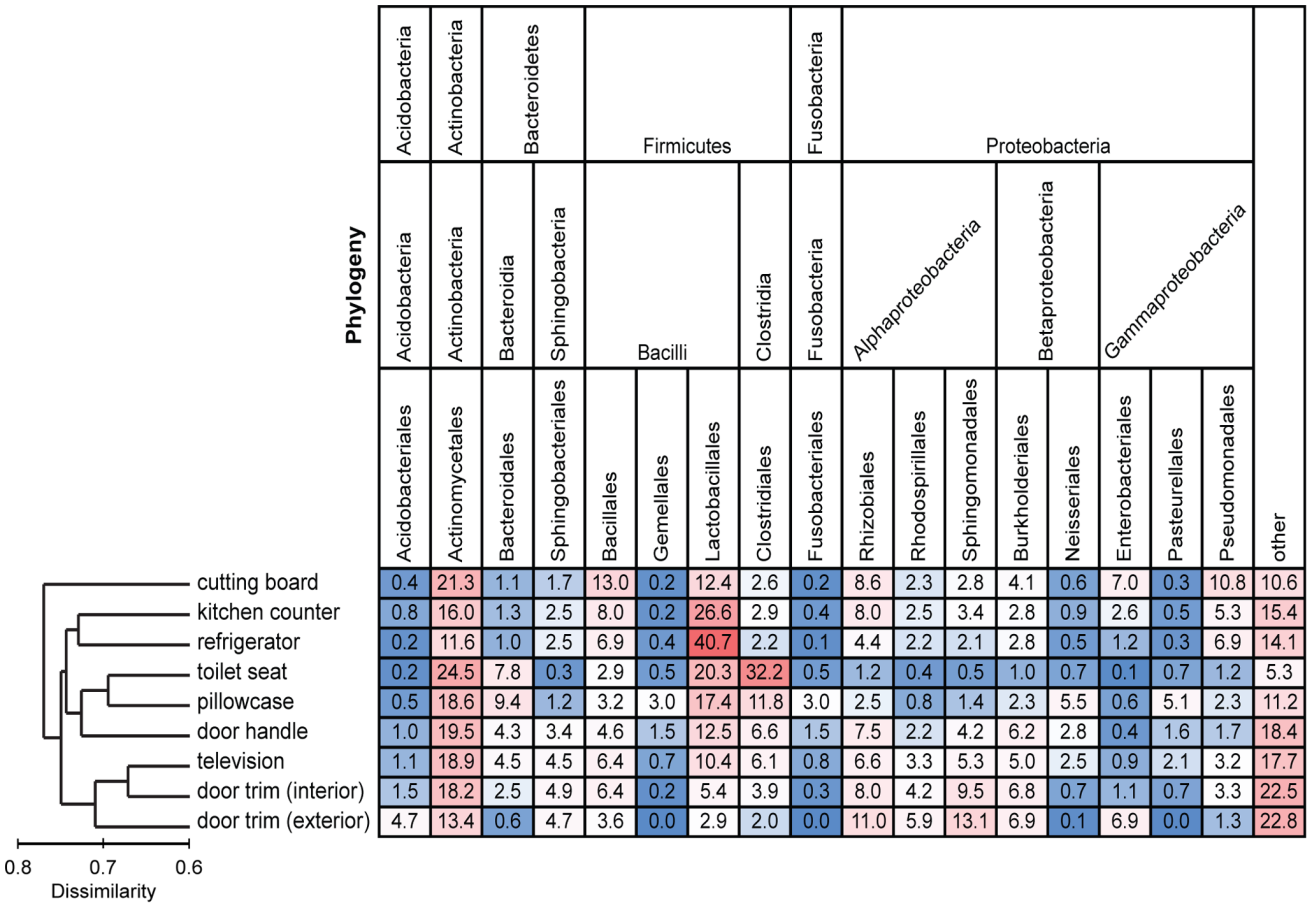
Heat map of the mean relative abundances of dominant bacterial taxa across the nine sampled locations. Each column is colored so that taxa with high relative abundances are red, intermediate relative abundances are white, and low abundances are blue. The dendrogram on the left summarizes the overall degree of dissimilarity (calculated from unweighted UniFrac values) of the bacterial communities in each of the sampled locations relative to each other. The dendrogram was created using mean UniFrac values for each of the sampled locations.

Across all of the collected samples, the dominant phyla (in terms of number of sequences) were Proteobacteria, Firmicutes, and Actinobacteria which represented 34%, 32%, and 20%, respectively, of all 16S rRNA gene sequences. The bacterial families with the highest relative abundances across all of the collected samples were the Streptococcaceae (8.9%), Corynebacteriaceae (5.6%), and Lactobacillaceae (5.1%). Bacterial community composition differed significantly between each sampling site across the 40 homes (*P*<0.05 for all pairwise comparisons). In general, samples clustered into the following general groups: depositional environments (both outdoor and indoor door trims, TV screen), kitchen-associated (cutting board, counter, refrigerator), and frequently touched surfaces (toilet seat, pillowcase, door handle) (Figure 2). The relative abundances of different taxa in relation to these clustering patterns are shown in Figure 3. For example, Lactobacillales (which include Streptococcaceae) were far less abundant in the door trim samples than in the other surfaces sampled. Members of the Clostridiales, Bacteroidales, Fusobacteriales, and Neisseriales orders typically made up a larger proportion of the bacterial sequences recovered from the toilet seat surfaces and pillowcases compared to other surfaces. Actinomycetales had consistently high relative abundances across all surfaces (12–25%), but Pseudomonadales taxa were relatively more abundant on surfaces found within kitchens. The communities on the door trims inside and outside the homes were distinct from one another with the outside samples having relatively higher abundances of Acidobacteria, Sphingomonadales, and Enterobacteriales taxa and lower relative abundances of taxa belonging to the Firmicutes phylum (Figure 3).

**Figure 4:**
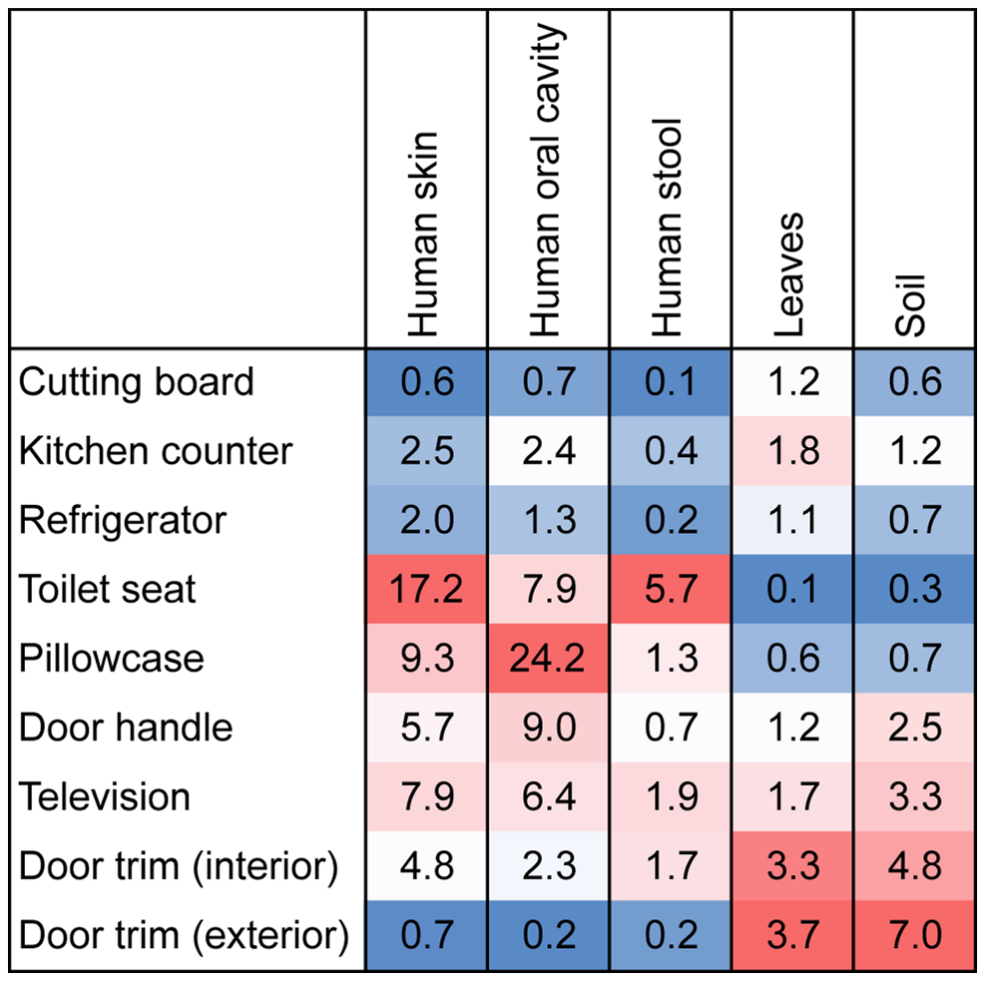
Source tracking analysis showing relative proportion of bacteria at each sampling site associated with given sources. Values represent median percentages. Warmer colors indicate greater influences of particular sources across the sites. As many of the taxa at a given site could not be attributed to individual sources and some of the sources had more indicator taxa than other sources, these results show changes in the relative importance of individual sources across sites, not comparisons across sources within sites. For example, these results show that soil is a more important source of bacteria on door trims than on cutting boards, but these results cannot be used to directly compare the relative importance of soil versus leaves as sources of bacteria at individual locations.

As we have previously analyzed a large number of samples from possible bacterial source environments inside and outside the home, including soil [43], human skin, mouth and stool [41], and the surfaces of leaves or produce items [19], [42], we used a source-tracking approach to quantify how differences in likely sources may have contributed to the distinct communities found on the different surfaces sampled. Using this approach, we identified the relative importance of different possible source environments as contributors to the bacteria found on each of the nine sampled surfaces (Figure 4). These analyses show that human-associated sources were important on many of the surfaces sampled, with human skin contributing to a relatively high proportion of the bacteria found in many of the interior surfaces including the toilet seat, pillowcase, television screen, door handle, and interior door trim (Figure 4). This pattern is exemplified by the high relative abundances of taxa commonly found on skin in these environments, including Streptococcaceae, Corynebacteriaceae, and Lactobacillaceae (Table S2). Bacteria from the human mouth and stool were important on the pillowcase and the toilet seat, respectively, while bacteria from leaves or produce were particularly abundant on the door trim surfaces and, to a lesser degree, the kitchen surfaces. Soil-derived bacteria were widespread, but found to be most abundant on the exterior door trim samples (Figure 4).

### Variation in Community Structure across Homes

As evident from Figure 2, the communities found at a given sampling location (habitat) varied in composition across the 40 homes. Although sampling location was the most important factor structuring the communities and each location harbored significantly distinct communities (as noted above), there was significant variability among homes for the individual sampling locations (*P* = 0.001). The between-home differences accounted for one-quarter to one-half as much variation as the location-specific differences within homes. This was particularly evident for the communities collected from the kitchen environments that were highly variable in composition across the homes (Figure 2).

Although we had limited data on the specific environmental conditions found within the homes, we could use collected data on house occupancy, location, and design to ascertain which of these factors contributed to the inter-home variability in community composition for the individual sampling locations. We found that this variation could be attributed, in part, to the presence of dogs in homes (*P = *0.001). In particular, the presence of dogs was associated with a different community composition on pillowcases and TV screens (*P*<0.005; Figure S2). The pillowcases and TV screens in homes with dogs had greater relative abundances of specific microbial taxa, including Porphyromonadaceae, Rhodocyclaceae, and Pasteurellaceae, that are also more abundant on dog fur than on human skin (Figure S3). This variation in community composition coincided with greater OTU richness at these locations in homes with dogs: on average, homes with dogs had 42% (*P* = 0.006) and 57% (*P* = 0.004) more OTUs on pillowcases and TV screens, respectively, compared to homes without pets.

We also found a significant relationship between exterior and interior microbial community composition (*P* = 0.006, rho = 0.30 for the door trim samples), suggesting that information on the types of bacteria found in the air outside the home can be used to predict, in part, the composition of the bacterial communities found within homes. We tested other factors, including number of human occupants, presence of cats, presence of children, use of pesticides, presence of carpet, and the presence of and types of allergies, but none of these factors had a significant influence on the bacterial communities at any of the sampling locations (P>0.05 in all cases). Either these factors were not important in structuring house-associated bacterial communities across the sampled locations or our sampling effort was insufficient to quantify the importance of those factors. For example, we could not detect an effect of cats on house-associated microbial communities; however, with only three homes having cats and no dogs, we may have lacked sufficient replication to detect such an effect. Likewise, we could not test the influence of other factors that may influence the structure of house-associated bacterial communities, including house environmental conditions (e.g., temperature, humidity), cleaning frequency, or specific surface materials, as we did not collect sufficient information on these factors from the homes sampled.

## Discussion

We share our homes with an enormous diversity of organisms, including many tens of animal species, plants, and, perhaps with the greatest consequences for our health and well-being, a broad diversity of bacteria and fungi. No home is without life, the question is simply which life occurs in a given home. Ideally, one would hope that we would manage the life in our homes so as to minimize the risk of harmful taxa and maximize the diversity of beneficial ones. However, before we do this, we first need to understand who is where and why. When it comes to microbial taxa, we are just taking this first step.

Not surprisingly, the bacterial diversity found in each of the nine individual locations was high with individual samples typically having more than 100 unique taxa. Similar diversity levels have been noted in previous studies of other house surfaces (e.g., [19]), and we are undoubtedly underestimating the diversity found within the habitats of homes that we sampled; the criterion we used to distinguish OTUs is conservative such that each OTU is likely to include multiple species and deeper sequencing would have allowed more rare taxa to be surveyed. However, while we encountered high levels of diversity in all habitats sampled, the median diversity of some of the communities, such as those found on door trim, was several times greater than that of others, such as cutting boards. *A priori* there do not exist clear theoretical predictions about the changes in bacterial diversity across habitats within homes. On the one hand, if neutral processes govern the diversity of microbial communities, we might expect habitats with high immigration rates and low local extinction rates (due to disturbance/cleaning) to have higher diversity (e.g., [46]). One could plausibly argue that door trim and television screens, the habitats in which we observed high microbial diversity, have a higher immigration rate due to the deposition of aerosolized bacteria with many members of these taxa remaining on the surfaces for extended periods of time due to infrequent cleaning. A similar pattern was reported in Flores et al. [19] who observed that depositional environments within kitchens that are not regularly cleaned typically harbor higher levels of diversity. On the other hand, it is also possible that deterministic processes govern the diversity of local habitats. Perhaps some environmental characteristics of door trim, and the other diverse habitats we encountered, allow more taxa to coexist. Our data do not allow us to distinguish these two possibilities, but suggest that studies of colonization/extinction rates (or temporal turnover) across house-associated habitats would be a fruitful avenue for future research.

More conspicuous than the differences in diversity among habitats within homes were the differences in bacterial community composition. Each of the nine sampled locations harbored significantly distinct communities with the variability within homes across the nine sampled locations being greater than the variability between homes for a given location. Other studies have also observed a high degree of spatial variability in the structure of bacterial communities found across different surfaces within kitchens [19], bathrooms [16], or offices [47]. Likewise, previous work has demonstrated that individual bacterial taxa often exhibit predictable distribution patterns across household surfaces [21]. The high degree of spatial variability in microbial community structure within the homes sampled here could be driven by differences in the type or frequency of disturbance, particularly as related to cleaning and cleaning products or differences in environmental conditions across the sampled locations. For example, changes in humidity, substrate type, temperature, and humidity could influence the composition of these surface-associated bacterial communities. Although our study design does not allow us to specifically identify which of these myriad of possible factors may be responsible for the observed patterns, our results do suggest that many of the intra-home differences and the clustering of the samples into the general groups (Figures 2 and 3) seem to be driven by differences in the sources of bacteria deposited onto the particular surfaces (Figure 4).

A number of the sampled habitats, including pillowcases and toilet seats, were dominated by skin-associated bacteria (Figure 4), a finding that is not surprising given that these surfaces regularly come into contact with exposed skin. Moreover, this finding provides further evidence that skin-associated bacteria are commonly dispersed throughout the built environment [16], [48], that such bacteria can survive on surfaces for extended periods of time [49], and that skin bacteria are more common on touched surfaces [19]. Other human-associated microbes were also common on the surfaces sampled; mouth-derived bacteria were common on pillowcases and fecal-derived bacteria were relatively abundant on the toilet seats (Figure 4). Together these results highlight that our bodies are the source for many of the bacteria found on surfaces within our homes and that our homes likely carry an identifiable signature of our own microbiomes. Furthermore, these results reinforce the idea that people sharing households also share a significant fraction of the bacterial taxa on and in their bodies [50]. Although there is a large body of literature examining how pathogens may be transferred between individuals from touched surfaces, these results suggest that the exchange of bacterial taxa between the house microbiome and the microbiome of its human occupants may be a more general phenomenon – involving thousands of taxa rather than just a few pathogens – and is worthy of more detailed study.

Kitchen-associated communities had appreciable abundances of bacteria sourced from food items brought into the kitchen as well as a number of bacterial taxa (including sphingomonads) common on other types of moist surfaces [18]. The taxa encountered here were similar to those described by Flores et al. [19] in an intensive study of many habitats in each of four kitchens. Interestingly, the kitchen counter and the refrigerator shared relatively similar communities even though these surfaces present very distinct environmental conditions. In this case, the shared sources of bacteria (or shared cleaning frequencies) may supersede any effects that local environmental conditions may have on bacterial community composition.

Compared to the samples collected from inside homes, the exterior door trim had far lower abundances of human-associated bacteria and appeared to be dominated by microbes commonly found in soil and on leaf surfaces (Figure 4). Both soil and leaf surfaces are common sources of aerosolized bacteria in the near-surface atmosphere [51] and many of the taxa found on the exterior door trim (including Sphingomonadales, Burkholderiales, and Pseudomonadales) have also been shown to be dominant in aerosol samples collected directly from outdoor air [52]–[54]. This apparent similarity between door trim samples and aerosol samples collected directly from the near-surface atmosphere suggests that door trim (or equivalent surfaces) could possibly be used as passive aerosol collectors to map microbial distributions in the atmosphere without the expense associated with deploying networks of specific aerosol collecting devices.

While strong differences across locations within houses exist and were consistent from one house to the next, some of the locations harbored bacterial communities that were highly variable in composition across the homes. A number of factors could account for this inter-home variability, including factors that we did not include in our analyses because they were either unmeasured (e.g., outdoor biodiversity [5]) or could not be resolved by sampling such a limited number of homes in one geographic region (e.g., regional climate). Some differences in how well individuals sampled particular substrates might also play a role. The net effect of such unmeasured factors should be to lead to unexplained variation among the homes we studied, variation that while potentially deterministic would be hard to account for in our analyses. Surprisingly, even without accounting for this variation, our results do provide some insight into the factors that account for this inter-home variation. For example, nearly all homes have some level of natural ventilation that would allow for outdoor microbes to gain entry into the home, and we did find evidence that the types of bacteria found outside the home influence the types found inside the home as there was a significant correlation between outer and inner bacterial communities across homes. Although ventilation rates were not quantified, we would expect this dispersal of bacterial taxa into homes to be higher in homes (or during seasons) when natural ventilation rates are highest [25]. Since the microbial diversity found in the outdoor atmosphere can be affected by a number of factors, including the surrounding land-use/vegetation type [52], we might expect house location to be an important factor influencing the types of microbes found within homes but no such effects were observed here. All the sampled homes were within an area of approximately 1000 km^2^ so any potential differences in airborne microbial taxa may be minimal if they are regionally driven. Future work would benefit from a broader sampling of homes across larger geographic regions to determine the extent to which the outside environment may shape the composition of those bacterial communities found within our homes.

We found that the presence of dogs in the house can have a large effect on the types of microbes found on household surfaces (Figures S2, S3) a phenomenon that has also been observed in other studies (e.g., [28]). The presence of dogs had significant effects on both the diversity and the types of bacteria found within homes on surfaces that may be in direct contact with dogs (e.g., pillows) and those that they are unlikely to ever touch (television screens). Furthermore, the taxa driving the differences between the household dog and non-dog communities seem to be derived directly from the dogs themselves (Figure S3). Although it was not our objective to determine the effects these dog-associated bacterial taxa may have on human health, it has long been suggested that individuals in homes with dogs might be less at risk for allergenic diseases. In particular, research suggests that pregnant mothers who live in houses with dogs are less likely to give birth to children who go on to develop allergies [55]–[57] or atopic dermatitis [28]. The early hypothesis linking allergies and dog presence was based on the untested assumption that houses with dogs tended to have different bacteria than those without. Building on previous work on this topic conducted by Fujimura et al. [28], our study provides evidence to robustly support this assumption. Not only does the presence of dogs influence the relative abundances of specific microbial taxa found within homes, dogs appear to be the main contributor to differences in microbial diversity across homes for selected locations. When you bring a dog into your house, you are not just bringing a dog, you are also introducing a suite of dog-associated taxa directly into your home environment, some of which may have direct or indirect effects on human health.

Our work highlights that our homes are composed of many distinct habitats that harbor distinct bacterial communities. The composition of these communities is likely driven by numerous factors, including the differential sources of bacteria dispersed onto the surfaces, and many of the community assembly patterns appear to be predictable. The microbial communities that live within homes clearly provide unique opportunities for testing ecological theories of community assembly, theories that have been derived almost exclusively from research on plant and animal communities. Practically speaking, however, the more significant result is our observation of strong differences in bacterial communities among houses, even for particular habitats. In our dataset, a single feature of the home environment (the presence of a dog) explained a large portion of the variability in bacterial community composition and diversity in some of the habitats sampled – but clearly there are other unmeasured or undetected factors that are likely important in structuring communities across homes. To the extent that the differences in household microbial communities are associated with differences in health and well-being, understanding these missing factors seems important. However, given the large number of potential explanatory variables, such work will require much larger sample sizes than were possible here.

## Supporting Information

Figure S1 **Diagram of the nine locations sampled within each of the 40 homes.** Insets emphasize the habitat characteristics of each sampling site.
(TIFF)

Figure S2 **Principle coordinates plots showing effect of dogs on bacterial community composition on (A) pillowcases and (B) TV screens.** Points closer together are more similar in terms of their bacterial composition.
(TIF)

Figure S3 **Presence of dogs in home influences relative abundance of bacterial taxa.** Differences in relative abundance of selected taxa between homes with dogs and those without pets on pillowcases and TV screens (A) and between dog fur and human skin (B; data from Song et al. [58]). The same taxa are more abundant in homes with dogs and on dogs relative to on humans.
(TIF)

Table S1 OTUs considered to be contaminants and removed prior to downstream analyses. OTU IDs and taxonomy labels are from the 97% similarity OTUs of the Greengenes February 2011 release.
(DOCX)

Table S2 Indicator taxa used for source tracking analysis.
(DOCX)
