# Supplementary figures and images for: Home Life: Factors Structuring the Bacterial Diversity Found within and between Homes
Source: PLoS One. 2013 May 22;8(5):e64133. doi: 10.1371/journal.pone.0064133 (PMC3661444; doi:10.1371/journal.pone.0064133)

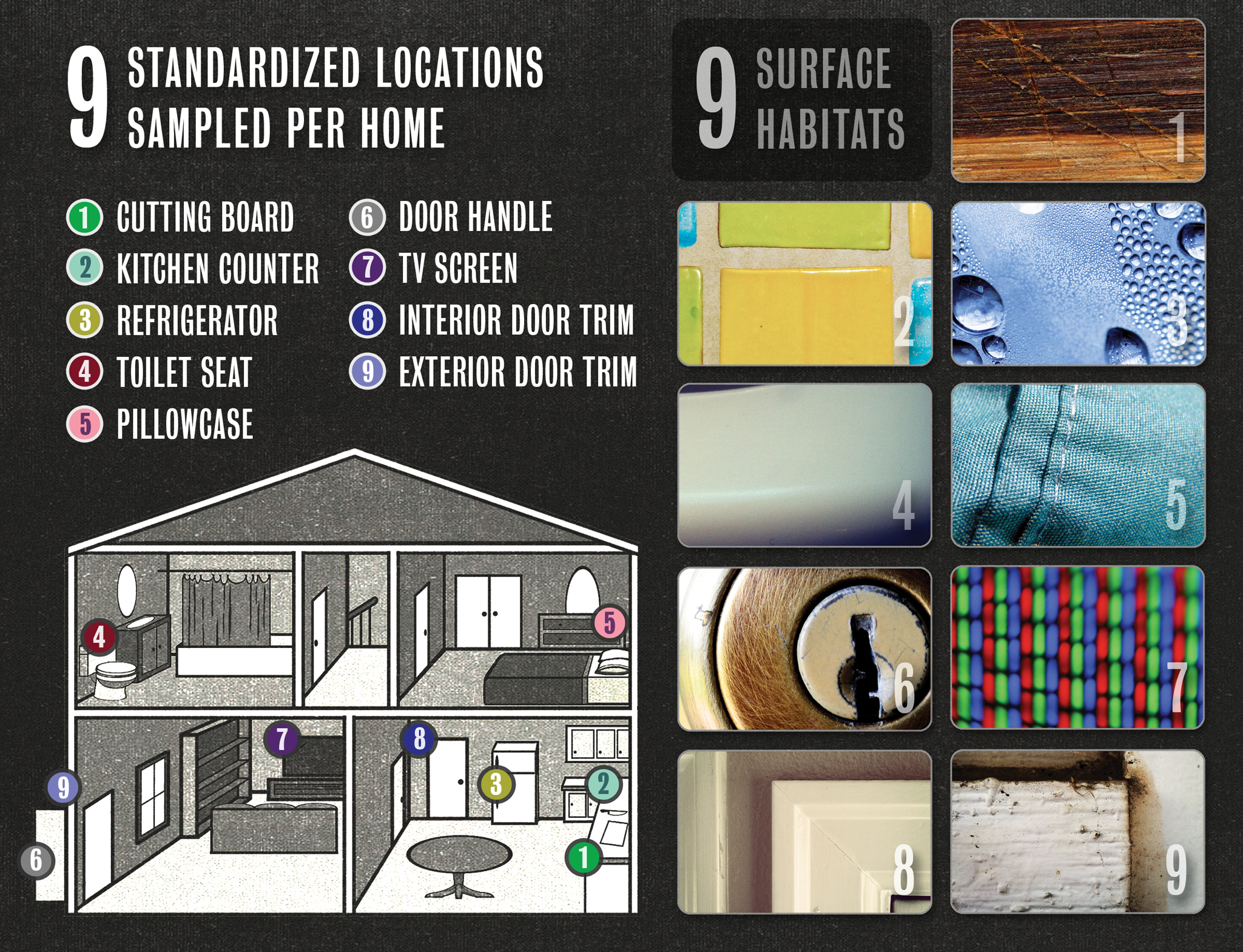

Supplement: Figure S1 — Diagram of the nine locations sampled within each of the 40 homes. Insets emphasize the habitat characteristics of each sampling site. (TIFF) [file pone.0064133.s001.tiff]

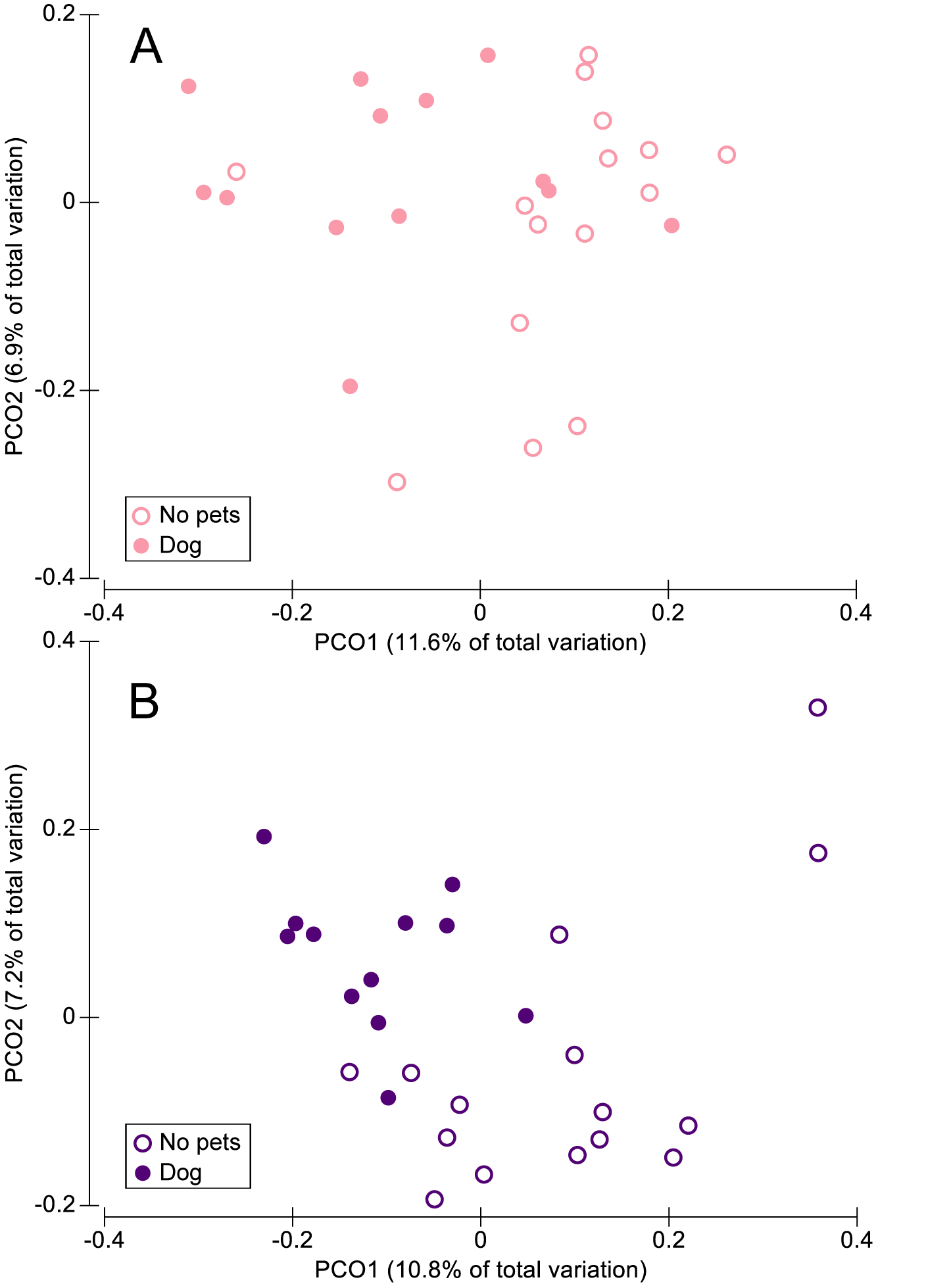

Supplement: Figure S2 — Principle coordinates plots showing effect of dogs on bacterial community composition on (A) pillowcases and (B) TV screens. Points closer together are more similar in terms of their bacterial composition. (TIF) [file pone.0064133.s002.tif]

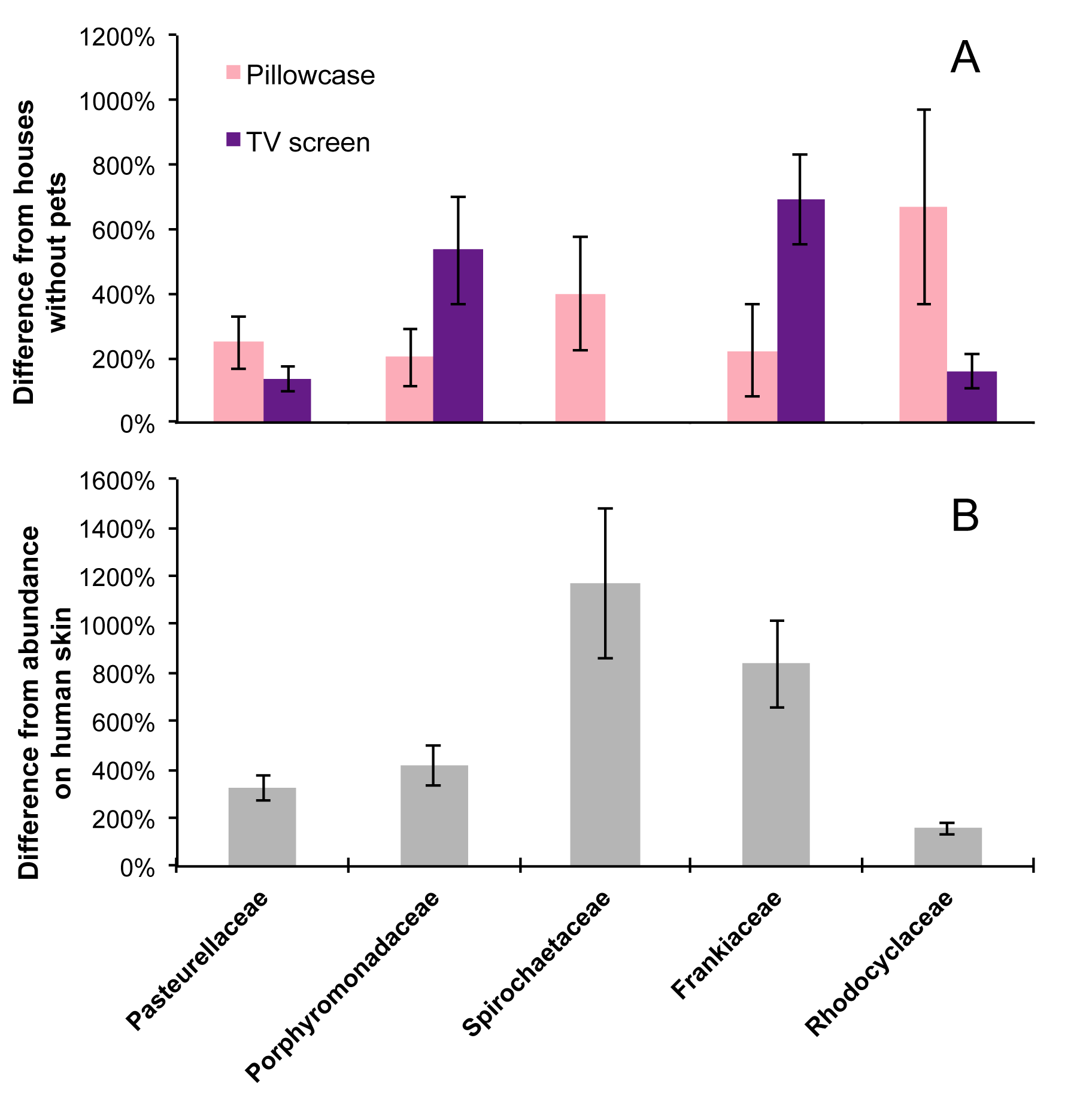

Supplement: Figure S3 — Presence of dogs in home influences relative abundance of bacterial taxa. Differences in relative abundance of selected taxa between homes with dogs and those without pets on pillowcases and TV screens (A) and between dog fur and human skin (B; data from Song et al. [58]). The same taxa are more abundant in homes with dogs and on dogs relative to on humans. (TIF) [file pone.0064133.s003.tif]
