# Supplementary material for: Home Life: Factors Structuring the Bacterial Diversity Found within and between Homes
Source: PLoS One. 2013 May 22;8(5):e64133. doi: 10.1371/journal.pone.0064133 (PMC3661444; doi:10.1371/journal.pone.0064133)
Supplement: Table S1 — OTUs considered to be contaminants and removed prior to downstream analyses. OTU IDs and taxonomy labels are from the 97% similarity OTUs of the Greengenes February 2011 release. (DOCX) [file pone.0064133.s004.docx]

| **OTU ID** | **Taxonomy** |
| --- | --- |
| 587313 | p__Acidobacteria; c__Acidobacteria; o__Acidobacteriales; f__; g__; s__ |
| 206192 | p__Acidobacteria; c__Acidobacteria; o__Acidobacteriales; f__; g__; s__ |
| 306018 | p__Acidobacteria; c__Acidobacteria; o__Acidobacteriales; f__; g__; s__ |
| 225522 | p__Acidobacteria; c__Acidobacteria; o__Acidobacteriales; f__; g__; s__ |
| 218467 | p__Acidobacteria; c__Acidobacteria; o__Acidobacteriales; f__; g__; s__ |
| 273309 | p__Acidobacteria; c__Acidobacteria; o__Acidobacteriales; f__; g__; s__ |
| 209723 | p__Acidobacteria; c__Acidobacteria; o__Acidobacteriales; f__; g__; s__ |
| 201720 | p__Actinobacteria; c__; o__; f__; g__; s__ |
| 320879 | p__Actinobacteria; c__Actinobacteria; o__Actinomycetales; f__Corynebacteriaceae; g__; s__ |
| 552026 | p__Actinobacteria; c__Actinobacteria; o__Actinomycetales; f__Corynebacteriaceae; g__Corynebacterium; s__ |
| 536902 | p__Actinobacteria; c__Actinobacteria; o__Actinomycetales; f__Corynebacteriaceae; g__Corynebacterium; s__ |
| 270102 | p__Actinobacteria; c__Actinobacteria; o__Actinomycetales; f__Corynebacteriaceae; g__Corynebacterium; s__ |
| 358127 | p__Actinobacteria; c__Actinobacteria; o__Actinomycetales; f__Corynebacteriaceae; g__Corynebacterium; s__ |
| 470178 | p__Actinobacteria; c__Actinobacteria; o__Actinomycetales; f__Corynebacteriaceae; g__Corynebacterium; s__Corynebacteriumaurimucosum |
| 470188 | p__Actinobacteria; c__Actinobacteria; o__Actinomycetales; f__Corynebacteriaceae; g__Corynebacterium; s__Corynebacteriumefficiens |
| 470213 | p__Actinobacteria; c__Actinobacteria; o__Actinomycetales; f__Corynebacteriaceae; g__Corynebacterium; s__Corynebacteriummatruchotii |
| 470219 | p__Actinobacteria; c__Actinobacteria; o__Actinomycetales; f__Corynebacteriaceae; g__Corynebacterium; s__Corynebacteriumtuberculostearicum |
| 272208 | p__Actinobacteria; c__Actinobacteria; o__Actinomycetales; f__Dietziaceae; g__Dietzia; s__Dietziamaris |
| 13321 | p__Actinobacteria; c__Actinobacteria; o__Actinomycetales; f__Gordoniaceae; g__Gordonia; s__ |
| 556066 | p__Actinobacteria; c__Actinobacteria; o__Actinomycetales; f__Microbacteriaceae; g__Agromyces; s__ |
| 79881 | p__Actinobacteria; c__Actinobacteria; o__Actinomycetales; f__Microbacteriaceae; g__Cryobacterium; s__Cryobacteriumpsychrophilum |
| 207157 | p__Actinobacteria; c__Actinobacteria; o__Actinomycetales; f__Microbacteriaceae; g__Leucobacter; s__ |
| 290069 | p__Actinobacteria; c__Actinobacteria; o__Actinomycetales; f__Micrococcaceae; g__Arthrobacter; s__ |
| 58301 | p__Actinobacteria; c__Actinobacteria; o__Actinomycetales; f__Micrococcaceae; g__Rothia; s__Rothiaaeria |
| 241543 | p__Actinobacteria; c__Actinobacteria; o__Actinomycetales; f__Nocardiaceae; g__Rhodococcus; s__ |
| 13534 | p__Actinobacteria; c__Actinobacteria; o__Actinomycetales; f__Nocardiaceae; g__Rhodococcus; s__ |
| 214610 | p__Actinobacteria; c__Actinobacteria; o__Actinomycetales; f__Nocardiaceae; g__Rhodococcus; s__ |
| 131262 | p__Actinobacteria; c__Actinobacteria; o__Actinomycetales; f__Nocardiaceae; g__Rhodococcus; s__ |
| 13581 | p__Actinobacteria; c__Actinobacteria; o__Actinomycetales; f__Nocardiaceae; g__Rhodococcus; s__Rhodococcusfascians |
| 13568 | p__Actinobacteria; c__Actinobacteria; o__Actinomycetales; f__Nocardiaceae; g__Rhodococcus; s__Rhodococcusgloberulus |
| 586825 | p__Actinobacteria; c__Actinobacteria; o__Actinomycetales; f__Nocardioidaceae; g__; s__ |
| 365828 | p__Actinobacteria; c__Actinobacteria; o__Actinomycetales; f__Nocardioidaceae; g__; s__ |
| 204284 | p__Actinobacteria; c__Actinobacteria; o__Actinomycetales; f__Nocardioidaceae; g__; s__ |
| 531515 | p__Actinobacteria; c__Actinobacteria; o__Actinomycetales; f__Nocardioidaceae; g__; s__ |
| 154684 | p__Actinobacteria; c__Actinobacteria; o__Actinomycetales; f__Nocardioidaceae; g__Pimelobacter; s__ |
| 368907 | p__Actinobacteria; c__Actinobacteria; o__Actinomycetales; f__Propionibacteriaceae; g__Propionibacterium; s__Propionibacteriumacnes |
| 199105 | p__Actinobacteria; c__Actinobacteria; o__Actinomycetales; f__Streptomycetaceae; g__Streptomyces; s__ |
| 204894 | p__Bacteroidetes; c__Bacteroidia; o__Bacteroidales; f__; g__; s__ |
| 549765 | p__Bacteroidetes; c__Bacteroidia; o__Bacteroidales; f__; g__; s__ |
| 76102 | p__Bacteroidetes; c__Bacteroidia; o__Bacteroidales; f__; g__; s__ |
| 569237 | p__Bacteroidetes; c__Bacteroidia; o__Bacteroidales; f__Prevotellaceae; g__; s__ |
| 103209 | p__Bacteroidetes; c__Bacteroidia; o__Bacteroidales; f__Prevotellaceae; g__Prevotella; s__ |
| 340840 | p__Bacteroidetes; c__Bacteroidia; o__Bacteroidales; f__Prevotellaceae; g__Prevotella; s__ |
| 516464 | p__Bacteroidetes; c__Bacteroidia; o__Bacteroidales; f__Prevotellaceae; g__Prevotella; s__ |
| 274839 | p__Bacteroidetes; c__Bacteroidia; o__Bacteroidales; f__Prevotellaceae; g__Prevotella; s__ |
| 178237 | p__Bacteroidetes; c__Flavobacteria; o__; f__; g__; s__ |
| 253429 | p__Bacteroidetes; c__Flavobacteria; o__Flavobacteriales; f__Flavobacteriaceae; g__; s__ |
| 115159 | p__Bacteroidetes; c__Flavobacteria; o__Flavobacteriales; f__Flavobacteriaceae; g__Chryseobacterium; s__ |
| 311868 | p__Bacteroidetes; c__Sphingobacteria; o__Sphingobacteriales; f__Flammeovirgaceae; g__; s__ |
| 252068 | p__Bacteroidetes; c__Sphingobacteria; o__Sphingobacteriales; f__Flammeovirgaceae; g__; s__ |
| 112775 | p__Bacteroidetes; c__Sphingobacteria; o__Sphingobacteriales; f__Flexibacteraceae; g__; s__ |
| 60254 | p__Cyanobacteria; c__Chloroplast; o__Streptophyta; f__; g__; s__ |
| 3549 | p__Cyanobacteria; c__Chloroplast; o__Streptophyta; f__; g__; s__ |
| 77182 | p__Firmicutes; c__Bacilli; o__Bacillales; f__Bacillaceae; g__; s__ |
| 206803 | p__Firmicutes; c__Bacilli; o__Bacillales; f__Bacillaceae; g__; s__ |
| 46071 | p__Firmicutes; c__Bacilli; o__Bacillales; f__Bacillaceae; g__; s__ |
| 252650 | p__Firmicutes; c__Bacilli; o__Bacillales; f__Bacillaceae; g__; s__ |
| 23244 | p__Firmicutes; c__Bacilli; o__Bacillales; f__Bacillaceae; g__Bacillus; s__ |
| 157637 | p__Firmicutes; c__Bacilli; o__Bacillales; f__Bacillaceae; g__Bacillus; s__ |
| 567864 | p__Firmicutes; c__Bacilli; o__Bacillales; f__Bacillaceae; g__Bacillus; s__ |
| 594063 | p__Firmicutes; c__Bacilli; o__Bacillales; f__Bacillaceae; g__Bacillus; s__ |
| 575425 | p__Firmicutes; c__Bacilli; o__Bacillales; f__Bacillaceae; g__Bacillus; s__Bacillusbadius |
| 252209 | p__Firmicutes; c__Bacilli; o__Bacillales; f__Paenibacillaceae; g__Paenibacillus; s__Paenibacillusamylolyticus |
| 378462 | p__Firmicutes; c__Bacilli; o__Bacillales; f__Staphylococcaceae; g__Staphylococcus; s__ |
| 269541 | p__Firmicutes; c__Bacilli; o__Bacillales; f__Staphylococcaceae; g__Staphylococcus; s__ |
| 578911 | p__Firmicutes; c__Bacilli; o__Exiguobacterales; f__Exiguobacteraceae; g__Exiguobacterium; s__ |
| 98605 | p__Firmicutes; c__Bacilli; o__Lactobacillales; f__Streptococcaceae; g__Streptococcus; s__Streptococcussanguinis |
| 306249 | p__Firmicutes; c__Clostridia; o__Clostridiales; f__Lachnospiraceae; g__; s__ |
| 355315 | p__Proteobacteria; c__Alphaproteobacteria; o__Caulobacterales; f__Caulobacteraceae; g__Brevundimonas; s__ |
| 573135 | p__Proteobacteria; c__Alphaproteobacteria; o__Rhizobiales; f__Bradyrhizobiaceae; g__; s__ |
| 100954 | p__Proteobacteria; c__Alphaproteobacteria; o__Rhizobiales; f__Bradyrhizobiaceae; g__Bradyrhizobium; s__ |
| 146948 | p__Proteobacteria; c__Alphaproteobacteria; o__Rhizobiales; f__Brucellaceae; g__Pseudochrobactrum; s__ |
| 565555 | p__Proteobacteria; c__Alphaproteobacteria; o__Rhizobiales; f__Hyphomicrobiaceae; g__Devosia; s__ |
| 335585 | p__Proteobacteria; c__Alphaproteobacteria; o__Rhizobiales; f__Methylobacteriaceae; g__Methylobacterium; s__ |
| 573545 | p__Proteobacteria; c__Alphaproteobacteria; o__Rhizobiales; f__Rhizobiaceae; g__Shinella; s__ |
| 527854 | p__Proteobacteria; c__Alphaproteobacteria; o__Rhodobacterales; f__Rhodobacteraceae; g__Paracoccus; s__ |
| 130803 | p__Proteobacteria; c__Alphaproteobacteria; o__Sphingomonadales; f__; g__; s__ |
| 162144 | p__Proteobacteria; c__Alphaproteobacteria; o__Sphingomonadales; f__; g__; s__ |
| 161477 | p__Proteobacteria; c__Alphaproteobacteria; o__Sphingomonadales; f__; g__; s__ |
| 354557 | p__Proteobacteria; c__Alphaproteobacteria; o__Sphingomonadales; f__Erythrobacteraceae; g__; s__ |
| 589380 | p__Proteobacteria; c__Alphaproteobacteria; o__Sphingomonadales; f__Sphingomonadaceae; g__; s__ |
| 217733 | p__Proteobacteria; c__Alphaproteobacteria; o__Sphingomonadales; f__Sphingomonadaceae; g__; s__ |
| 92490 | p__Proteobacteria; c__Alphaproteobacteria; o__Sphingomonadales; f__Sphingomonadaceae; g__Novosphingobium; s__ |
| 192248 | p__Proteobacteria; c__Alphaproteobacteria; o__Sphingomonadales; f__Sphingomonadaceae; g__Novosphingobium; s__ |
| 182669 | p__Proteobacteria; c__Alphaproteobacteria; o__Sphingomonadales; f__Sphingomonadaceae; g__Novosphingobium; s__ |
| 38548 | p__Proteobacteria; c__Alphaproteobacteria; o__Sphingomonadales; f__Sphingomonadaceae; g__Novosphingobium; s__ |
| 435628 | p__Proteobacteria; c__Alphaproteobacteria; o__Sphingomonadales; f__Sphingomonadaceae; g__Novosphingobium; s__ |
| 360253 | p__Proteobacteria; c__Alphaproteobacteria; o__Sphingomonadales; f__Sphingomonadaceae; g__Novosphingobium; s__ |
| 89574 | p__Proteobacteria; c__Alphaproteobacteria; o__Sphingomonadales; f__Sphingomonadaceae; g__Novosphingobium; s__ |
| 180839 | p__Proteobacteria; c__Alphaproteobacteria; o__Sphingomonadales; f__Sphingomonadaceae; g__Novosphingobium; s__ |
| 6195 | p__Proteobacteria; c__Alphaproteobacteria; o__Sphingomonadales; f__Sphingomonadaceae; g__Sphingobium; s__ |
| 370761 | p__Proteobacteria; c__Alphaproteobacteria; o__Sphingomonadales; f__Sphingomonadaceae; g__Sphingobium; s__ |
| 312453 | p__Proteobacteria; c__Alphaproteobacteria; o__Sphingomonadales; f__Sphingomonadaceae; g__Sphingomonas; s__ |
| 319666 | p__Proteobacteria; c__Alphaproteobacteria; o__Sphingomonadales; f__Sphingomonadaceae; g__Sphingomonas; s__ |
| 158370 | p__Proteobacteria; c__Alphaproteobacteria; o__Sphingomonadales; f__Sphingomonadaceae; g__Sphingomonas; s__ |
| 560773 | p__Proteobacteria; c__Alphaproteobacteria; o__Sphingomonadales; f__Sphingomonadaceae; g__Sphingomonas; s__Sphingomonasasaccharolytica |
| 228077 | p__Proteobacteria; c__Betaproteobacteria; o__; f__; g__; s__ |
| 557460 | p__Proteobacteria; c__Betaproteobacteria; o__Burkholderiales; f__; g__; s__ |
| 233054 | p__Proteobacteria; c__Betaproteobacteria; o__Burkholderiales; f__; g__; s__ |
| 101552 | p__Proteobacteria; c__Betaproteobacteria; o__Burkholderiales; f__; g__Aquabacterium; s__ |
| 217417 | p__Proteobacteria; c__Betaproteobacteria; o__Burkholderiales; f__; g__Methylibium; s__ |
| 342757 | p__Proteobacteria; c__Betaproteobacteria; o__Burkholderiales; f__; g__Methylibium; s__ |
| 234716 | p__Proteobacteria; c__Betaproteobacteria; o__Burkholderiales; f__; g__Methylibium; s__ |
| 64874 | p__Proteobacteria; c__Betaproteobacteria; o__Burkholderiales; f__Burkholderiaceae; g__; s__ |
| 33919 | p__Proteobacteria; c__Betaproteobacteria; o__Burkholderiales; f__Burkholderiaceae; g__Burkholderia; s__ |
| 150508 | p__Proteobacteria; c__Betaproteobacteria; o__Burkholderiales; f__Burkholderiaceae; g__Burkholderia; s__ |
| 139689 | p__Proteobacteria; c__Betaproteobacteria; o__Burkholderiales; f__Burkholderiaceae; g__Burkholderia; s__ |
| 111824 | p__Proteobacteria; c__Betaproteobacteria; o__Burkholderiales; f__Burkholderiaceae; g__Burkholderia; s__Burkholderiagraminis |
| 243860 | p__Proteobacteria; c__Betaproteobacteria; o__Burkholderiales; f__Burkholderiaceae; g__Ralstonia; s__ |
| 349239 | p__Proteobacteria; c__Betaproteobacteria; o__Burkholderiales; f__Burkholderiaceae; g__Ralstonia; s__ |
| 293030 | p__Proteobacteria; c__Betaproteobacteria; o__Burkholderiales; f__Burkholderiaceae; g__Ralstonia; s__ |
| 552978 | p__Proteobacteria; c__Betaproteobacteria; o__Burkholderiales; f__Comamonadaceae; g__; s__ |
| 589483 | p__Proteobacteria; c__Betaproteobacteria; o__Burkholderiales; f__Comamonadaceae; g__; s__ |
| 211171 | p__Proteobacteria; c__Betaproteobacteria; o__Burkholderiales; f__Comamonadaceae; g__; s__ |
| 86368 | p__Proteobacteria; c__Betaproteobacteria; o__Burkholderiales; f__Comamonadaceae; g__; s__ |
| 547824 | p__Proteobacteria; c__Betaproteobacteria; o__Burkholderiales; f__Comamonadaceae; g__; s__ |
| 155949 | p__Proteobacteria; c__Betaproteobacteria; o__Burkholderiales; f__Comamonadaceae; g__; s__ |
| 213853 | p__Proteobacteria; c__Betaproteobacteria; o__Burkholderiales; f__Comamonadaceae; g__; s__ |
| 141254 | p__Proteobacteria; c__Betaproteobacteria; o__Burkholderiales; f__Comamonadaceae; g__; s__ |
| 7037 | p__Proteobacteria; c__Betaproteobacteria; o__Burkholderiales; f__Comamonadaceae; g__; s__ |
| 74685 | p__Proteobacteria; c__Betaproteobacteria; o__Burkholderiales; f__Comamonadaceae; g__Acidovorax; s__ |
| 143519 | p__Proteobacteria; c__Betaproteobacteria; o__Burkholderiales; f__Comamonadaceae; g__Acidovorax; s__ |
| 141709 | p__Proteobacteria; c__Betaproteobacteria; o__Burkholderiales; f__Comamonadaceae; g__Acidovorax; s__ |
| 592118 | p__Proteobacteria; c__Betaproteobacteria; o__Burkholderiales; f__Comamonadaceae; g__Acidovorax; s__Acidovoraxdelafieldii |
| 59902 | p__Proteobacteria; c__Betaproteobacteria; o__Burkholderiales; f__Comamonadaceae; g__Aquamonas; s__ |
| 368509 | p__Proteobacteria; c__Betaproteobacteria; o__Burkholderiales; f__Comamonadaceae; g__Aquamonas; s__Aquamonasfontana |
| 576010 | p__Proteobacteria; c__Betaproteobacteria; o__Burkholderiales; f__Comamonadaceae; g__Comamonas; s__ |
| 310843 | p__Proteobacteria; c__Betaproteobacteria; o__Burkholderiales; f__Comamonadaceae; g__Hylemonella; s__ |
| 156790 | p__Proteobacteria; c__Betaproteobacteria; o__Burkholderiales; f__Comamonadaceae; g__Polaromonas; s__Polaromonassp.JS666 |
| 76621 | p__Proteobacteria; c__Betaproteobacteria; o__Burkholderiales; f__Comamonadaceae; g__Ramlibacter; s__ |
| 113128 | p__Proteobacteria; c__Betaproteobacteria; o__Burkholderiales; f__Comamonadaceae; g__Ramlibacter; s__ |
| 228981 | p__Proteobacteria; c__Betaproteobacteria; o__Burkholderiales; f__Comamonadaceae; g__Rhodoferax; s__ |
| 391129 | p__Proteobacteria; c__Betaproteobacteria; o__Burkholderiales; f__Comamonadaceae; g__Variovorax; s__Variovoraxparadoxus |
| 278496 | p__Proteobacteria; c__Betaproteobacteria; o__Burkholderiales; f__Oxalobacteraceae; g__; s__ |
| 570154 | p__Proteobacteria; c__Betaproteobacteria; o__Burkholderiales; f__Oxalobacteraceae; g__; s__ |
| 537594 | p__Proteobacteria; c__Betaproteobacteria; o__Burkholderiales; f__Oxalobacteraceae; g__; s__ |
| 157564 | p__Proteobacteria; c__Betaproteobacteria; o__Burkholderiales; f__Oxalobacteraceae; g__; s__ |
| 277795 | p__Proteobacteria; c__Betaproteobacteria; o__Burkholderiales; f__Oxalobacteraceae; g__; s__ |
| 181996 | p__Proteobacteria; c__Betaproteobacteria; o__Burkholderiales; f__Oxalobacteraceae; g__Herbaspirillum; s__ |
| 7303 | p__Proteobacteria; c__Betaproteobacteria; o__Burkholderiales; f__Oxalobacteraceae; g__Herbaspirillum; s__ |
| 313175 | p__Proteobacteria; c__Betaproteobacteria; o__Burkholderiales; f__Oxalobacteraceae; g__Janthinobacterium; s__Janthinobacteriumlividum |
| 102382 | p__Proteobacteria; c__Betaproteobacteria; o__Burkholderiales; f__Oxalobacteraceae; g__Massilia; s__Massiliatimonae |
| 155597 | p__Proteobacteria; c__Betaproteobacteria; o__Burkholderiales; f__Oxalobacteraceae; g__Massilia; s__Massiliatimonae |
| 155215 | p__Proteobacteria; c__Betaproteobacteria; o__Rhodocyclales; f__Rhodocyclaceae; g__Methyloversatilis; s__ |
| 104408 | p__Proteobacteria; c__Betaproteobacteria; o__Rhodocyclales; f__Rhodocyclaceae; g__Zoogloea; s__ |
| 136353 | p__Proteobacteria; c__Gammaproteobacteria; o__; f__; g__; s__ |
| 388951 | p__Proteobacteria; c__Gammaproteobacteria; o__; f__; g__; s__ |
| 254988 | p__Proteobacteria; c__Gammaproteobacteria; o__; f__; g__; s__ |
| 101021 | p__Proteobacteria; c__Gammaproteobacteria; o__Aeromonadales; f__; g__; s__ |
| 168069 | p__Proteobacteria; c__Gammaproteobacteria; o__Aeromonadales; f__Aeromonadaceae; g__Aeromonas; s__ |
| 576068 | p__Proteobacteria; c__Gammaproteobacteria; o__Aeromonadales; f__Aeromonadaceae; g__Aeromonas; s__ |
| 9349 | p__Proteobacteria; c__Gammaproteobacteria; o__Aeromonadales; f__Succinivibrionaceae; g__Ruminobacter; s__ |
| 38578 | p__Proteobacteria; c__Gammaproteobacteria; o__Alteromonadales; f__; g__Alkalimonas; s__ |
| 163714 | p__Proteobacteria; c__Gammaproteobacteria; o__Alteromonadales; f__; g__Alkalimonas; s__ |
| 207297 | p__Proteobacteria; c__Gammaproteobacteria; o__Chromatiales; f__; g__; s__ |
| 538780 | p__Proteobacteria; c__Gammaproteobacteria; o__Chromatiales; f__; g__; s__ |
| 539039 | p__Proteobacteria; c__Gammaproteobacteria; o__Chromatiales; f__; g__; s__ |
| 575230 | p__Proteobacteria; c__Gammaproteobacteria; o__Chromatiales; f__; g__; s__ |
| 253074 | p__Proteobacteria; c__Gammaproteobacteria; o__Chromatiales; f__; g__; s__ |
| 7621 | p__Proteobacteria; c__Gammaproteobacteria; o__Chromatiales; f__; g__; s__ |
| 7596 | p__Proteobacteria; c__Gammaproteobacteria; o__Chromatiales; f__Thiotrichaceae; g__Achromatium; s__Achromatiumoxaliferum |
| 175189 | p__Proteobacteria; c__Gammaproteobacteria; o__Enterobacteriales; f__Enterobacteriaceae; g__; s__ |
| 287789 | p__Proteobacteria; c__Gammaproteobacteria; o__Enterobacteriales; f__Enterobacteriaceae; g__; s__ |
| 166908 | p__Proteobacteria; c__Gammaproteobacteria; o__Enterobacteriales; f__Enterobacteriaceae; g__; s__ |
| 9727 | p__Proteobacteria; c__Gammaproteobacteria; o__Enterobacteriales; f__Enterobacteriaceae; g__; s__ |
| 288710 | p__Proteobacteria; c__Gammaproteobacteria; o__Enterobacteriales; f__Enterobacteriaceae; g__; s__ |
| 236821 | p__Proteobacteria; c__Gammaproteobacteria; o__Enterobacteriales; f__Enterobacteriaceae; g__; s__ |
| 292134 | p__Proteobacteria; c__Gammaproteobacteria; o__Enterobacteriales; f__Enterobacteriaceae; g__; s__ |
| 368849 | p__Proteobacteria; c__Gammaproteobacteria; o__Enterobacteriales; f__Enterobacteriaceae; g__; s__ |
| 202356 | p__Proteobacteria; c__Gammaproteobacteria; o__Enterobacteriales; f__Enterobacteriaceae; g__; s__ |
| 10309 | p__Proteobacteria; c__Gammaproteobacteria; o__Enterobacteriales; f__Enterobacteriaceae; g__; s__ |
| 429887 | p__Proteobacteria; c__Gammaproteobacteria; o__Enterobacteriales; f__Enterobacteriaceae; g__; s__ |
| 142545 | p__Proteobacteria; c__Gammaproteobacteria; o__Enterobacteriales; f__Enterobacteriaceae; g__; s__ |
| 10115 | p__Proteobacteria; c__Gammaproteobacteria; o__Enterobacteriales; f__Enterobacteriaceae; g__; s__ |
| 561320 | p__Proteobacteria; c__Gammaproteobacteria; o__Enterobacteriales; f__Enterobacteriaceae; g__; s__ |
| 531978 | p__Proteobacteria; c__Gammaproteobacteria; o__Enterobacteriales; f__Enterobacteriaceae; g__; s__ |
| 10113 | p__Proteobacteria; c__Gammaproteobacteria; o__Enterobacteriales; f__Enterobacteriaceae; g__; s__ |
| 9838 | p__Proteobacteria; c__Gammaproteobacteria; o__Enterobacteriales; f__Enterobacteriaceae; g__; s__ |
| 291008 | p__Proteobacteria; c__Gammaproteobacteria; o__Enterobacteriales; f__Enterobacteriaceae; g__; s__ |
| 248470 | p__Proteobacteria; c__Gammaproteobacteria; o__Enterobacteriales; f__Enterobacteriaceae; g__; s__symbiontofNoeetapupillata |
| 246330 | p__Proteobacteria; c__Gammaproteobacteria; o__Enterobacteriales; f__Enterobacteriaceae; g__Cronobacter; s__ |
| 569973 | p__Proteobacteria; c__Gammaproteobacteria; o__Enterobacteriales; f__Enterobacteriaceae; g__Cronobacter; s__ |
| 537651 | p__Proteobacteria; c__Gammaproteobacteria; o__Enterobacteriales; f__Enterobacteriaceae; g__Erwinia; s__ |
| 9812 | p__Proteobacteria; c__Gammaproteobacteria; o__Enterobacteriales; f__Enterobacteriaceae; g__Erwinia; s__Erwiniatoletana |
| 302025 | p__Proteobacteria; c__Gammaproteobacteria; o__Enterobacteriales; f__Enterobacteriaceae; g__Escherichia; s__ |
| 240711 | p__Proteobacteria; c__Gammaproteobacteria; o__Enterobacteriales; f__Enterobacteriaceae; g__Escherichia; s__ |
| 295395 | p__Proteobacteria; c__Gammaproteobacteria; o__Enterobacteriales; f__Enterobacteriaceae; g__Escherichia; s__ |
| 169182 | p__Proteobacteria; c__Gammaproteobacteria; o__Enterobacteriales; f__Enterobacteriaceae; g__Escherichia; s__ |
| 537192 | p__Proteobacteria; c__Gammaproteobacteria; o__Enterobacteriales; f__Enterobacteriaceae; g__Escherichia; s__ |
| 173654 | p__Proteobacteria; c__Gammaproteobacteria; o__Enterobacteriales; f__Enterobacteriaceae; g__Escherichia; s__ |
| 592346 | p__Proteobacteria; c__Gammaproteobacteria; o__Enterobacteriales; f__Enterobacteriaceae; g__Klebsiella; s__ |
| 307981 | p__Proteobacteria; c__Gammaproteobacteria; o__Enterobacteriales; f__Enterobacteriaceae; g__Klebsiella; s__ |
| 557905 | p__Proteobacteria; c__Gammaproteobacteria; o__Enterobacteriales; f__Enterobacteriaceae; g__Pantoea; s__ |
| 218720 | p__Proteobacteria; c__Gammaproteobacteria; o__Enterobacteriales; f__Enterobacteriaceae; g__Pantoea; s__ |
| 534898 | p__Proteobacteria; c__Gammaproteobacteria; o__Enterobacteriales; f__Enterobacteriaceae; g__Pantoea; s__Pantoeaananatis |
| 168031 | p__Proteobacteria; c__Gammaproteobacteria; o__Enterobacteriales; f__Enterobacteriaceae; g__Pantoea; s__Pantoeaananatis |
| 152527 | p__Proteobacteria; c__Gammaproteobacteria; o__Enterobacteriales; f__Enterobacteriaceae; g__Pantoea; s__Pantoeaoleae |
| 167440 | p__Proteobacteria; c__Gammaproteobacteria; o__Enterobacteriales; f__Enterobacteriaceae; g__Plesiomonas; s__Plesiomonasshigelloides |
| 560314 | p__Proteobacteria; c__Gammaproteobacteria; o__Enterobacteriales; f__Enterobacteriaceae; g__Plesiomonas; s__Plesiomonasshigelloides |
| 255774 | p__Proteobacteria; c__Gammaproteobacteria; o__Enterobacteriales; f__Enterobacteriaceae; g__Rahnella; s__ |
| 565501 | p__Proteobacteria; c__Gammaproteobacteria; o__Enterobacteriales; f__Enterobacteriaceae; g__Raoultella; s__ |
| 329096 | p__Proteobacteria; c__Gammaproteobacteria; o__Enterobacteriales; f__Enterobacteriaceae; g__Raoultella; s__ |
| 222043 | p__Proteobacteria; c__Gammaproteobacteria; o__Enterobacteriales; f__Enterobacteriaceae; g__Raoultella; s__Raoultellaornithinolytica |
| 213522 | p__Proteobacteria; c__Gammaproteobacteria; o__Enterobacteriales; f__Enterobacteriaceae; g__Serratia; s__ |
| 10189 | p__Proteobacteria; c__Gammaproteobacteria; o__Enterobacteriales; f__Enterobacteriaceae; g__Serratia; s__ |
| 225626 | p__Proteobacteria; c__Gammaproteobacteria; o__Enterobacteriales; f__Enterobacteriaceae; g__Serratia; s__ |
| 240135 | p__Proteobacteria; c__Gammaproteobacteria; o__Enterobacteriales; f__Enterobacteriaceae; g__Serratia; s__ |
| 555692 | p__Proteobacteria; c__Gammaproteobacteria; o__Enterobacteriales; f__Enterobacteriaceae; g__Serratia; s__ |
| 206221 | p__Proteobacteria; c__Gammaproteobacteria; o__Enterobacteriales; f__Enterobacteriaceae; g__Serratia; s__ |
| 554574 | p__Proteobacteria; c__Gammaproteobacteria; o__Enterobacteriales; f__Enterobacteriaceae; g__Serratia; s__ |
| 583608 | p__Proteobacteria; c__Gammaproteobacteria; o__Enterobacteriales; f__Enterobacteriaceae; g__Serratia; s__CandidatusSerratiasymbiotica |
| 10167 | p__Proteobacteria; c__Gammaproteobacteria; o__Enterobacteriales; f__Enterobacteriaceae; g__Serratia; s__Serratiaentomophila |
| 332958 | p__Proteobacteria; c__Gammaproteobacteria; o__Enterobacteriales; f__Enterobacteriaceae; g__Serratia; s__Serratiamarcescens |
| 262838 | p__Proteobacteria; c__Gammaproteobacteria; o__Enterobacteriales; f__Enterobacteriaceae; g__Serratia; s__Serratiamarcescens |
| 161584 | p__Proteobacteria; c__Gammaproteobacteria; o__Enterobacteriales; f__Enterobacteriaceae; g__Yersinia; s__ |
| 274754 | p__Proteobacteria; c__Gammaproteobacteria; o__Enterobacteriales; f__Enterobacteriaceae; g__Yersinia; s__ |
| 10390 | p__Proteobacteria; c__Gammaproteobacteria; o__Enterobacteriales; f__Enterobacteriaceae; g__Yersinia; s__ |
| 16391 | p__Proteobacteria; c__Gammaproteobacteria; o__Legionellales; f__Coxiellaceae; g__Rickettsiella; s__ |
| 73506 | p__Proteobacteria; c__Gammaproteobacteria; o__Legionellales; f__Coxiellaceae; g__Rickettsiella; s__ |
| 113578 | p__Proteobacteria; c__Gammaproteobacteria; o__Methylococcales; f__; g__; s__ |
| 275378 | p__Proteobacteria; c__Gammaproteobacteria; o__Oceanospirillales; f__Alcanivoracaceae; g__Alcanivorax; s__ |
| 114493 | p__Proteobacteria; c__Gammaproteobacteria; o__Oceanospirillales; f__Oceanospirillaceae; g__Marinomonas; s__Marinomonasaquimarina |
| 145318 | p__Proteobacteria; c__Gammaproteobacteria; o__Oceanospirillales; f__Oceanospirillaceae; g__Marinomonas; s__Marinomonassp.MED121 |
| 152723 | p__Proteobacteria; c__Gammaproteobacteria; o__Oceanospirillales; f__Saccharospirillaceae; g__Reinekea; s__Reinekeablandensis |
| 140393 | p__Proteobacteria; c__Gammaproteobacteria; o__Pasteurellales; f__Pasteurellaceae; g__; s__BisgaardTaxon10 |
| 94166 | p__Proteobacteria; c__Gammaproteobacteria; o__Pasteurellales; f__Pasteurellaceae; g__Haemophilus; s__Haemophilusparainfluenzae |
| 286210 | p__Proteobacteria; c__Gammaproteobacteria; o__Pasteurellales; f__Pasteurellaceae; g__Pasteurella; s__Pasteurellamultocida |
| 197845 | p__Proteobacteria; c__Gammaproteobacteria; o__Pseudomonadales; f__; g__; s__ |
| 484436 | p__Proteobacteria; c__Gammaproteobacteria; o__Pseudomonadales; f__Moraxellaceae; g__; s__ |
| 143131 | p__Proteobacteria; c__Gammaproteobacteria; o__Pseudomonadales; f__Moraxellaceae; g__; s__ |
| 346926 | p__Proteobacteria; c__Gammaproteobacteria; o__Pseudomonadales; f__Moraxellaceae; g__Acinetobacter; s__ |
| 194641 | p__Proteobacteria; c__Gammaproteobacteria; o__Pseudomonadales; f__Moraxellaceae; g__Acinetobacter; s__ |
| 103428 | p__Proteobacteria; c__Gammaproteobacteria; o__Pseudomonadales; f__Moraxellaceae; g__Acinetobacter; s__ |
| 584176 | p__Proteobacteria; c__Gammaproteobacteria; o__Pseudomonadales; f__Moraxellaceae; g__Acinetobacter; s__ |
| 361751 | p__Proteobacteria; c__Gammaproteobacteria; o__Pseudomonadales; f__Moraxellaceae; g__Acinetobacter; s__ |
| 569668 | p__Proteobacteria; c__Gammaproteobacteria; o__Pseudomonadales; f__Moraxellaceae; g__Acinetobacter; s__ |
| 30013 | p__Proteobacteria; c__Gammaproteobacteria; o__Pseudomonadales; f__Moraxellaceae; g__Acinetobacter; s__ |
| 222902 | p__Proteobacteria; c__Gammaproteobacteria; o__Pseudomonadales; f__Moraxellaceae; g__Acinetobacter; s__ |
| 8400 | p__Proteobacteria; c__Gammaproteobacteria; o__Pseudomonadales; f__Moraxellaceae; g__Acinetobacter; s__ |
| 553964 | p__Proteobacteria; c__Gammaproteobacteria; o__Pseudomonadales; f__Moraxellaceae; g__Acinetobacter; s__ |
| 353461 | p__Proteobacteria; c__Gammaproteobacteria; o__Pseudomonadales; f__Moraxellaceae; g__Acinetobacter; s__ |
| 60258 | p__Proteobacteria; c__Gammaproteobacteria; o__Pseudomonadales; f__Moraxellaceae; g__Acinetobacter; s__Acinetobacterguillouiae |
| 249191 | p__Proteobacteria; c__Gammaproteobacteria; o__Pseudomonadales; f__Moraxellaceae; g__Acinetobacter; s__Acinetobacterjohnsonii |
| 535160 | p__Proteobacteria; c__Gammaproteobacteria; o__Pseudomonadales; f__Moraxellaceae; g__Acinetobacter; s__Acinetobacterjohnsonii |
| 317252 | p__Proteobacteria; c__Gammaproteobacteria; o__Pseudomonadales; f__Moraxellaceae; g__Acinetobacter; s__Acinetobacterjohnsonii |
| 254162 | p__Proteobacteria; c__Gammaproteobacteria; o__Pseudomonadales; f__Moraxellaceae; g__Acinetobacter; s__Acinetobacterjohnsonii |
| 356287 | p__Proteobacteria; c__Gammaproteobacteria; o__Pseudomonadales; f__Moraxellaceae; g__Acinetobacter; s__Acinetobacterjohnsonii |
| 216324 | p__Proteobacteria; c__Gammaproteobacteria; o__Pseudomonadales; f__Moraxellaceae; g__Acinetobacter; s__Acinetobacterjohnsonii |
| 351148 | p__Proteobacteria; c__Gammaproteobacteria; o__Pseudomonadales; f__Moraxellaceae; g__Acinetobacter; s__Acinetobacterjohnsonii |
| 242603 | p__Proteobacteria; c__Gammaproteobacteria; o__Pseudomonadales; f__Moraxellaceae; g__Acinetobacter; s__Acinetobacterjohnsonii |
| 159711 | p__Proteobacteria; c__Gammaproteobacteria; o__Pseudomonadales; f__Moraxellaceae; g__Acinetobacter; s__Acinetobacterlwoffii |
| 587004 | p__Proteobacteria; c__Gammaproteobacteria; o__Pseudomonadales; f__Moraxellaceae; g__Acinetobacter; s__Acinetobacterlwoffii |
| 471124 | p__Proteobacteria; c__Gammaproteobacteria; o__Pseudomonadales; f__Moraxellaceae; g__Acinetobacter; s__Acinetobacterradioresistens |
| 103620 | p__Proteobacteria; c__Gammaproteobacteria; o__Pseudomonadales; f__Moraxellaceae; g__Acinetobacter; s__Acinetobactersp.ADP1 |
| 39819 | p__Proteobacteria; c__Gammaproteobacteria; o__Pseudomonadales; f__Moraxellaceae; g__Acinetobacter; s__Acinetobacterursingii |
| 278666 | p__Proteobacteria; c__Gammaproteobacteria; o__Pseudomonadales; f__Moraxellaceae; g__Alkanindiges; s__ |
| 277352 | p__Proteobacteria; c__Gammaproteobacteria; o__Pseudomonadales; f__Moraxellaceae; g__Psychrobacter; s__ |
| 269774 | p__Proteobacteria; c__Gammaproteobacteria; o__Pseudomonadales; f__Moraxellaceae; g__Psychrobacter; s__ |
| 268361 | p__Proteobacteria; c__Gammaproteobacteria; o__Pseudomonadales; f__Moraxellaceae; g__Psychrobacter; s__ |
| 263567 | p__Proteobacteria; c__Gammaproteobacteria; o__Pseudomonadales; f__Pseudomonadaceae; g__; s__ |
| 133961 | p__Proteobacteria; c__Gammaproteobacteria; o__Pseudomonadales; f__Pseudomonadaceae; g__Pseudomonas; s__ |
| 269930 | p__Proteobacteria; c__Gammaproteobacteria; o__Pseudomonadales; f__Pseudomonadaceae; g__Pseudomonas; s__ |
| 25543 | p__Proteobacteria; c__Gammaproteobacteria; o__Pseudomonadales; f__Pseudomonadaceae; g__Pseudomonas; s__ |
| 141206 | p__Proteobacteria; c__Gammaproteobacteria; o__Pseudomonadales; f__Pseudomonadaceae; g__Pseudomonas; s__ |
| 571439 | p__Proteobacteria; c__Gammaproteobacteria; o__Pseudomonadales; f__Pseudomonadaceae; g__Pseudomonas; s__ |
| 242070 | p__Proteobacteria; c__Gammaproteobacteria; o__Pseudomonadales; f__Pseudomonadaceae; g__Pseudomonas; s__ |
| 393265 | p__Proteobacteria; c__Gammaproteobacteria; o__Pseudomonadales; f__Pseudomonadaceae; g__Pseudomonas; s__ |
| 140880 | p__Proteobacteria; c__Gammaproteobacteria; o__Pseudomonadales; f__Pseudomonadaceae; g__Pseudomonas; s__ |
| 400315 | p__Proteobacteria; c__Gammaproteobacteria; o__Pseudomonadales; f__Pseudomonadaceae; g__Pseudomonas; s__ |
| 289441 | p__Proteobacteria; c__Gammaproteobacteria; o__Pseudomonadales; f__Pseudomonadaceae; g__Pseudomonas; s__ |
| 103728 | p__Proteobacteria; c__Gammaproteobacteria; o__Pseudomonadales; f__Pseudomonadaceae; g__Pseudomonas; s__ |
| 414568 | p__Proteobacteria; c__Gammaproteobacteria; o__Pseudomonadales; f__Pseudomonadaceae; g__Pseudomonas; s__ |
| 264673 | p__Proteobacteria; c__Gammaproteobacteria; o__Pseudomonadales; f__Pseudomonadaceae; g__Pseudomonas; s__ |
| 410048 | p__Proteobacteria; c__Gammaproteobacteria; o__Pseudomonadales; f__Pseudomonadaceae; g__Pseudomonas; s__ |
| 202340 | p__Proteobacteria; c__Gammaproteobacteria; o__Pseudomonadales; f__Pseudomonadaceae; g__Pseudomonas; s__ |
| 110621 | p__Proteobacteria; c__Gammaproteobacteria; o__Pseudomonadales; f__Pseudomonadaceae; g__Pseudomonas; s__ |
| 327694 | p__Proteobacteria; c__Gammaproteobacteria; o__Pseudomonadales; f__Pseudomonadaceae; g__Pseudomonas; s__ |
| 398604 | p__Proteobacteria; c__Gammaproteobacteria; o__Pseudomonadales; f__Pseudomonadaceae; g__Pseudomonas; s__ |
| 394796 | p__Proteobacteria; c__Gammaproteobacteria; o__Pseudomonadales; f__Pseudomonadaceae; g__Pseudomonas; s__ |
| 223417 | p__Proteobacteria; c__Gammaproteobacteria; o__Pseudomonadales; f__Pseudomonadaceae; g__Pseudomonas; s__ |
| 139289 | p__Proteobacteria; c__Gammaproteobacteria; o__Pseudomonadales; f__Pseudomonadaceae; g__Pseudomonas; s__ |
| 260790 | p__Proteobacteria; c__Gammaproteobacteria; o__Pseudomonadales; f__Pseudomonadaceae; g__Pseudomonas; s__ |
| 411142 | p__Proteobacteria; c__Gammaproteobacteria; o__Pseudomonadales; f__Pseudomonadaceae; g__Pseudomonas; s__ |
| 65400 | p__Proteobacteria; c__Gammaproteobacteria; o__Pseudomonadales; f__Pseudomonadaceae; g__Pseudomonas; s__ |
| 272189 | p__Proteobacteria; c__Gammaproteobacteria; o__Pseudomonadales; f__Pseudomonadaceae; g__Pseudomonas; s__ |
| 276867 | p__Proteobacteria; c__Gammaproteobacteria; o__Pseudomonadales; f__Pseudomonadaceae; g__Pseudomonas; s__ |
| 140966 | p__Proteobacteria; c__Gammaproteobacteria; o__Pseudomonadales; f__Pseudomonadaceae; g__Pseudomonas; s__ |
| 283193 | p__Proteobacteria; c__Gammaproteobacteria; o__Pseudomonadales; f__Pseudomonadaceae; g__Pseudomonas; s__ |
| 155221 | p__Proteobacteria; c__Gammaproteobacteria; o__Pseudomonadales; f__Pseudomonadaceae; g__Pseudomonas; s__ |
| 280369 | p__Proteobacteria; c__Gammaproteobacteria; o__Pseudomonadales; f__Pseudomonadaceae; g__Pseudomonas; s__ |
| 332073 | p__Proteobacteria; c__Gammaproteobacteria; o__Pseudomonadales; f__Pseudomonadaceae; g__Pseudomonas; s__ |
| 141127 | p__Proteobacteria; c__Gammaproteobacteria; o__Pseudomonadales; f__Pseudomonadaceae; g__Pseudomonas; s__ |
| 253171 | p__Proteobacteria; c__Gammaproteobacteria; o__Pseudomonadales; f__Pseudomonadaceae; g__Pseudomonas; s__ |
| 558838 | p__Proteobacteria; c__Gammaproteobacteria; o__Pseudomonadales; f__Pseudomonadaceae; g__Pseudomonas; s__ |
| 549107 | p__Proteobacteria; c__Gammaproteobacteria; o__Pseudomonadales; f__Pseudomonadaceae; g__Pseudomonas; s__ |
| 130002 | p__Proteobacteria; c__Gammaproteobacteria; o__Pseudomonadales; f__Pseudomonadaceae; g__Pseudomonas; s__ |
| 308767 | p__Proteobacteria; c__Gammaproteobacteria; o__Pseudomonadales; f__Pseudomonadaceae; g__Pseudomonas; s__ |
| 129642 | p__Proteobacteria; c__Gammaproteobacteria; o__Pseudomonadales; f__Pseudomonadaceae; g__Pseudomonas; s__ |
| 237173 | p__Proteobacteria; c__Gammaproteobacteria; o__Pseudomonadales; f__Pseudomonadaceae; g__Pseudomonas; s__ |
| 593655 | p__Proteobacteria; c__Gammaproteobacteria; o__Pseudomonadales; f__Pseudomonadaceae; g__Pseudomonas; s__ |
| 78485 | p__Proteobacteria; c__Gammaproteobacteria; o__Pseudomonadales; f__Pseudomonadaceae; g__Pseudomonas; s__ |
| 315411 | p__Proteobacteria; c__Gammaproteobacteria; o__Pseudomonadales; f__Pseudomonadaceae; g__Pseudomonas; s__ |
| 195370 | p__Proteobacteria; c__Gammaproteobacteria; o__Pseudomonadales; f__Pseudomonadaceae; g__Pseudomonas; s__ |
| 263856 | p__Proteobacteria; c__Gammaproteobacteria; o__Pseudomonadales; f__Pseudomonadaceae; g__Pseudomonas; s__ |
| 108449 | p__Proteobacteria; c__Gammaproteobacteria; o__Pseudomonadales; f__Pseudomonadaceae; g__Pseudomonas; s__ |
| 25337 | p__Proteobacteria; c__Gammaproteobacteria; o__Pseudomonadales; f__Pseudomonadaceae; g__Pseudomonas; s__ |
| 109056 | p__Proteobacteria; c__Gammaproteobacteria; o__Pseudomonadales; f__Pseudomonadaceae; g__Pseudomonas; s__ |
| 140933 | p__Proteobacteria; c__Gammaproteobacteria; o__Pseudomonadales; f__Pseudomonadaceae; g__Pseudomonas; s__ |
| 350105 | p__Proteobacteria; c__Gammaproteobacteria; o__Pseudomonadales; f__Pseudomonadaceae; g__Pseudomonas; s__ |
| 193843 | p__Proteobacteria; c__Gammaproteobacteria; o__Pseudomonadales; f__Pseudomonadaceae; g__Pseudomonas; s__ |
| 318906 | p__Proteobacteria; c__Gammaproteobacteria; o__Pseudomonadales; f__Pseudomonadaceae; g__Pseudomonas; s__ |
| 340095 | p__Proteobacteria; c__Gammaproteobacteria; o__Pseudomonadales; f__Pseudomonadaceae; g__Pseudomonas; s__Pseudomonasstutzeri |
| 279231 | p__Proteobacteria; c__Gammaproteobacteria; o__Pseudomonadales; f__Pseudomonadaceae; g__Pseudomonas; s__Pseudomonasumsongensis |
| 270842 | p__Proteobacteria; c__Gammaproteobacteria; o__Pseudomonadales; f__Pseudomonadaceae; g__Pseudomonas; s__Pseudomonasumsongensis |
| 140765 | p__Proteobacteria; c__Gammaproteobacteria; o__Pseudomonadales; f__Pseudomonadaceae; g__Pseudomonas; s__Pseudomonasumsongensis |
| 144968 | p__Proteobacteria; c__Gammaproteobacteria; o__Pseudomonadales; f__Pseudomonadaceae; g__Pseudomonas; s__Pseudomonasveronii |
| 89393 | p__Proteobacteria; c__Gammaproteobacteria; o__Pseudomonadales; f__Pseudomonadaceae; g__Pseudomonas; s__Pseudomonasviridiflava |
| 179854 | p__Proteobacteria; c__Gammaproteobacteria; o__Pseudomonadales; f__Pseudomonadaceae; g__Pseudomonas; s__Pseudomonasviridiflava |
| 362563 | p__Proteobacteria; c__Gammaproteobacteria; o__Vibrionales; f__Vibrionaceae; g__Vibrio; s__ |
| 128050 | p__Proteobacteria; c__Gammaproteobacteria; o__Vibrionales; f__Vibrionaceae; g__Vibrio; s__ |
| 84937 | p__Proteobacteria; c__Gammaproteobacteria; o__Vibrionales; f__Vibrionaceae; g__Vibrio; s__Vibriogallicus |
| 217221 | p__Proteobacteria; c__Gammaproteobacteria; o__Xanthomonadales; f__Xanthomonadaceae; g__Pseudoxanthomonas; s__ |
| 275171 | p__Proteobacteria; c__Gammaproteobacteria; o__Xanthomonadales; f__Xanthomonadaceae; g__Stenotrophomonas; s__ |
| 389700 | p__Proteobacteria; c__Gammaproteobacteria; o__Xanthomonadales; f__Xanthomonadaceae; g__Stenotrophomonas; s__ |
| 593848 | p__Proteobacteria; c__Gammaproteobacteria; o__Xanthomonadales; f__Xanthomonadaceae; g__Stenotrophomonas; s__ |
| 429048 | p__Proteobacteria; c__Gammaproteobacteria; o__Xanthomonadales; f__Xanthomonadaceae; g__Stenotrophomonas; s__ |
| 516809 | p__Proteobacteria; c__Gammaproteobacteria; o__Xanthomonadales; f__Xanthomonadaceae; g__Stenotrophomonas; s__ |
| 259059 | p__Proteobacteria; c__Gammaproteobacteria; o__Xanthomonadales; f__Xanthomonadaceae; g__Stenotrophomonas; s__ |
| 85823 | p__Proteobacteria; c__Gammaproteobacteria; o__Xanthomonadales; f__Xanthomonadaceae; g__Xanthomonas; s__ |
| 3826 | p__Spirochaetes; c__Spirochaetes; o__Spirochaetales; f__Spirochaetaceae; g__Treponema; s__ |
| 103814 | p__Spirochaetes; c__Spirochaetes; o__Spirochaetales; f__Spirochaetaceae; g__Treponema; s__ |
| 304059 | p__Tenericutes; c__Erysipelotrichi; o__Erysipelotrichales; f__vadinHA31; g__RFN20; s__ |
| 49164 | p__Tenericutes; c__Mollicutes; o__Mycoplasmatales; f__Mycoplasmataceae; g__Mycoplasma; s__ |
| 209237 | p__Tenericutes; c__Mollicutes; o__Mycoplasmatales; f__Mycoplasmataceae; g__Mycoplasma; s__CandidatusMycoplasmahaemobos |
| 114673 | p__Tenericutes; c__Mollicutes; o__Mycoplasmatales; f__Mycoplasmataceae; g__Mycoplasma; s__Mycoplasmawenyonii |
| 301864 | p__Verrucomicrobia; c__Verruco-5; o__RFP12; f__; g__; s__ |
