# Supplementary material for: Home Life: Factors Structuring the Bacterial Diversity Found within and between Homes
Source: PLoS One. 2013 May 22;8(5):e64133. doi: 10.1371/journal.pone.0064133 (PMC3661444; doi:10.1371/journal.pone.0064133)
Supplement: Table S2 — Indicator taxa used for source tracking analysis. (DOCX) [file pone.0064133.s005.docx]

|  |  | **Indicator value** | **Median relative abundance (%)** | | | | |
| --- | --- | --- | --- | --- | --- | --- | --- |
| **Source** | **Indicator taxon** |  | Human skin | Human oral cavity | Human stool | Leaf | Soil |
| Human skin | Propionibacteriaceae | 0.99 | 37.2 | 0.0 | 0.0 | 0.0 | 0.0 |
|  | Staphylococcaceae | 0.94 | 2.6 | 0.0 | 0.0 | 0.0 | 0.0 |
|  | Corynebacteriaceae | 0.80 | 3.2 | 0.0 | 0.0 | 0.0 | 0.0 |
| Human oral cavity | Pasteurellaceae | 0.91 | 0.4 | 14.4 | 0.0 | 0.0 | 0.0 |
|  | Fusobacteriaceae | 0.86 | 0.0 | 2.0 | 0.0 | 0.0 | 0.0 |
|  | Veillonellaceae | 0.82 | 0.4 | 12.0 | 1.2 | 0.0 | 0.0 |
|  | Neisseriaceae | 0.74 | 0.4 | 5.0 | 0.0 | 0.0 | 0.0 |
|  | Campylobacteraceae | 0.68 | 0.0 | 0.8 | 0.0 | 0.0 | 0.0 |
|  | Leptotrichiaceae | 0.62 | 0.0 | 0.8 | 0.0 | 0.0 | 0.0 |
|  | Actinomycetaceae | 0.62 | 0.4 | 1.2 | 0.0 | 0.0 | 0.0 |
|  | Prevotellaceae | 0.61 | 0.0 | 8.4 | 0.0 | 0.0 | 0.0 |
| Human stool | Bacteroidaceae | 0.99 | 0.0 | 0.0 | 36.8 | 0.0 | 0.0 |
|  | Rikenellaceae | 0.95 | 0.0 | 0.0 | 2.4 | 0.0 | 0.0 |
|  | Ruminococcaceae | 0.95 | 0.0 | 0.0 | 11.6 | 0.0 | 0.0 |
|  | Lachnospiraceae | 0.83 | 0.0 | 1.6 | 10.4 | 0.0 | 0.0 |
| Leaf | Enterobacteriaceae | 0.85 | 0.4 | 0.0 | 0.0 | 15.2 | 0.0 |
|  | Flexibacteraceae | 0.73 | 0.0 | 0.0 | 0.0 | 3.6 | 0.0 |
|  | Deinococcaceae | 0.64 | 0.0 | 0.0 | 0.0 | 2.0 | 0.0 |
| Soil | Acidobacteria | 0.69 | 1.6 | 1.8 | 1.6 | 2.4 | 22.8 |
|  | Bradyrhizobiaceae | 0.79 | 0.0 | 0.0 | 0.0 | 0.0 | 2.0 |
|  | Hyphomicrobiaceae | 0.73 | 0.0 | 0.0 | 0.0 | 0.0 | 2.8 |
|  | Sinobacteraceae | 0.66 | 0.0 | 0.0 | 0.0 | 0.0 | 1.2 |
